# Review on the Processing and Properties of Polymer Nanocomposites and Nanocoatings and Their Applications in the Packaging, Automotive and Solar Energy Fields

**DOI:** 10.3390/nano7040074

**Published:** 2017-03-31

**Authors:** Kerstin Müller, Elodie Bugnicourt, Marcos Latorre, Maria Jorda, Yolanda Echegoyen Sanz, José M. Lagaron, Oliver Miesbauer, Alvise Bianchin, Steve Hankin, Uwe Bölz, Germán Pérez, Marius Jesdinszki, Martina Lindner, Zuzana Scheuerer, Sara Castelló, Markus Schmid

**Affiliations:** 1 Fraunhofer Institute for Process Engineering and Packaging IVV, Giggenhauser Strasse 35, 85354 Freising, Germany; kerstin.mueller@ivv.fraunhofer.de (K.M.); oliver.miesbauer@ivv.fraunhofer.de (O.M.); marius.jesdinszki@ivv.fraunhofer.de (M.J.); martina.lindner@ivv.fraunhofer.de (M.L.); zuzana.scheuerer@ivv.fraunhofer.de (Z.S.); 2 IRIS, Parc Mediterrani de la Tecnologia, Avda. Carl Friedrich Gauss 11, 08860 Castelldefels, Barcelona, Spain; ebugnicourt@iris.cat; 3 ITENE Instituto Tecnológico del Embalaje, Transporte y Logística, Albert Einstein, 1, 46980 Paterna, Spain; marcos.latorre@itene.com (M.L.); mjorda@itene.com (M.J.); 4 Institute of Agrochemistry and Food Technology (IATA)-CSIC, Avda. Agustín Escardino, 7, 46980 Paterna, Spain; yoesanz@uv.es (Y.E.S.); lagaron@iata.csic.es (J.M.L.); 5 Science Education Department, Facultat de Magisteri, Universitat de València, 46022 València, Spain; 6 MBN Nanomaterialia, via Bortolan 42, 31040 Vascon di Carbonera, Italy; research@mbn.it; 7 Institute of Occupational Medicine, Research Avenue North, Riccarton, Edinburgh, EH14 4AP, UK; steve.hankin@iom-world.org; 8 HPX Polymers GmbH, Ziegeleistraße 1, 82327 Tutzing, Germany; info@hpx-polymers.de; 9 Eurecat, Av. Universitat Autònoma 23, 08290 Cerdanyola del Vallès, Barcelona, Spain; german.perez@eurecat.org; 10 Bioinicia, Calle Algepser, 65-Nave 3 | Polígono Industrial Táctica | 46980 Paterna (Valencia), Spain; sara.castello@bioinicia.com; 11 Chair for Food Packaging Technology, Technische Universität München, Weihenstephaner Steig 22, 85354 Freising, Germany

**Keywords:** nanodeposit, nanocomposite, electrospraying, barrier improvement, self-cleaning surfaces, light-weight materials

## Abstract

For the last decades, nanocomposites materials have been widely studied in the scientific literature as they provide substantial properties enhancements, even at low nanoparticles content. Their performance depends on a number of parameters but the nanoparticles dispersion and distribution state remains the key challenge in order to obtain the full nanocomposites’ potential in terms of, e.g., flame retardance, mechanical, barrier and thermal properties, etc., that would allow extending their use in the industry. While the amount of existing research and indeed review papers regarding the formulation of nanocomposites is already significant, after listing the most common applications, this review focuses more in-depth on the properties and materials of relevance in three target sectors: packaging, solar energy and automotive. In terms of advances in the processing of nanocomposites, this review discusses various enhancement technologies such as the use of ultrasounds for in-process nanoparticles dispersion. In the case of nanocoatings, it describes the different conventionally used processes as well as nanoparticles deposition by electro-hydrodynamic processing. All in all, this review gives the basics both in terms of composition and of processing aspects to reach optimal properties for using nanocomposites in the selected applications. As an outlook, up-to-date nanosafety issues are discussed.

## 1. Introduction

For the last decades, nanocomposites materials have been widely reported in the scientific literature to provide substantial properties enhancements, even at low nanoparticles content. In nanotechnology, polymer nanocomposites are defined as solids consisting of a mixture of two or more phase separated materials, where one or more dispersed phase is in nanoscale and a polymeric major phase. Materials can be referred to as nanoscaled when their size, meaning at least one of the three external dimensions range from approximately 1 nm to 100 nm [1]. Nanocomposites can be processed by conventional wet and dry processing techniques, yet in adjusted conditions vs. their neat counterparts. Polymer nanocomposites and nanoparticles can also be applied as nanocoatings, meaning a deposited nanoscale layer on selected substrates to reach specific surface behaviour [2,3]. 

There are a number of nanoparticles that have been reported to be used in the formulation of nanocomposites and which definition can be consulted in many extensive prior literature [4]. Those are generally divided in fibres (1D) platelets (2D) or particles (3D) depending on the number of dimensions they display in the nanoscale [5] and they generally differ from the microparticles commonly used in the composite sectors by a greater surface area. Among the polymeric matrix nanocomposites, since they are readily industrially available and low cost, nanoclays are among the most studied scientifically but are also the object of a number of commercial trials since the first work categorized as nanocomposites from Toyota leading to using nylon 6-clay hybrids in car equipment in 1989 [6]. Nano-oxides like TiO_2_, ZnO, SiO_2_ are also extensively used in the literature and commercial applications to provide respectively for example self-cleaning properties, UV protection or tailored rheological behaviour. Carbon nanotubes and more recently Graphene are gaining greater attention since their excellent intrinsic properties and unique structures open up new prospects as their production processes become more efficient, leading to greater availability and lower costs. 

The nanocomposite performance depends on a number of nanoparticles features such as the size, aspect ratio, specific surface area, volume fraction used, compatibility with the matrix and dispersion. In fact, although a long time has gone in the nanocomposites’ era, the dispersion state of nanoparticles remains the key challenge in order to obtain the full potential of properties enhancement (flame retardance, mechanical, barrier, thermal properties, etc.) at lower filler loading than for microcomposites. Not only can the nanoparticles themselves explain the observed effects, the impact of the interface between the matrix and particle also plays a very important role. Indeed, the extremely high surface area leads to change in the macromolecular state around the nanoparticles (e.g., composition gradient, crystallinity, changed mobility, etc.) that modifies the overall material behaviour [7]. 

The nanoparticles dispersion can be characterized by different states at nano-, micro- and macroscopic scales. For example, nanoclay based composites can show three different types of morphology: immiscible (e.g., microscale dispersion, tactoid), intercalated or exfoliated (miscible) composites [8]. The affinity between matrix and filler increases from tactoid over intercalated to exfoliated clays [9]. 

The dispersion and nanocoating thickness are generally characterized off line through the use of electronic microscopy [10,11], X-ray diffraction, etc. [12]. As opposed to standard composites, nanocomposites present the advantage to be potentially transparent although the optical properties can be highly affected by the nanocomposite morphology.

Several strategies have been used to improve dispersion quality, including either chemical or physical approaches. Surface modification to enhance the compatibility of the matrix and fillers is often used, for example through the grafting of organosilanes, or through the use of long chains alkyl ammonium clay platelets intercalating ions [13,14]. Alternatively, when applicable, in situ polymerization may be preferred to reach a good dispersion state that is sometimes difficult to reach when processing nanocomposites in highly viscous media [15,16]. 

In terms of physical methods, besides the use of mechanical mixing methods (high speed mixer, extruder, etc.), the application of ultrasonic vibrations has been reported to be effective in enhancing the dispersion state of nanoparticles both in solutions and melt polymers [17]. Electromagnetic fields application or high shear [18]/compression in one direction are also reported methods that can help in orientating the nanoparticles to create a structure that can, for example, maximize the gas tortuosity to limit the permeation across a packaging, reduce the flammability or increase the mechanical properties in a given direction.

Nanocoatings in turns allow maximizing the concentration in nanofillers on the surface of a material to create a specific effect while requiring a lower amount of nanoparticles than when dispersed in the bulk. 

Nanocoatings can be applied by different technologies such electrospray that can apply nanoparticles-based layers as opposed to established processes such as chemical or physical vapour deposition that rather allow the application of a continuous layer on an atomic scale. Electrospray present the advantage to work at atmospheric pressure and is therefore easily integrated in continuous production lines. Nanocoatings allow surface functionalisation to provide specific properties such as antimicrobial [19], self-healing [20], flame retardant [21], gas barrier [22], which are of interest for textiles [23], solar panels [24], packaging [22] and automotive fields [25] among others.

This review gives an insight on the application of nanocomposites and nanocoatings with a special focus on their prospects for:

- food and cosmetic packaging
- solar energy, especially organic photovoltaics
- automotive structural parts

In terms of packaging, different properties can be enhanced through the use of nanocomposites such as the gas barrier, antimicrobial properties, etc. The most frequently tested nanofillers are nanoclays montmorillonite (MMT) and kaolinite, carbon nanotubes (CNT) and graphene nanoplates (GNP) [26]. The improvement of barrier properties could more especially benefit biopolymers which generally have limited intrinsic barrier properties. Additionally, surface coatings can be used for modulating surface affinity of the packaging towards different liquids and pastes, e.g., to obtain water repellent paper-based packaging [27] or easy-to-empty features [28].

In terms of automotive applications, mechanical and electronic properties, thermal isolation, wear resistance and flame retardance are of interest among other improvements that can be provided by nanocomposites. In terms of mechanical reinforcement, whereas standard toughening additives and fillers normally lead to opposite variation of toughness and stiffness, the specific effect of nanoparticles allowing to improve both can benefit in the design of parts that comply similar structural functions at lower weight than micro-composites or neat polymers. Lighter but stronger materials allow optimal fuel consumption and increased safety.

In terms of solar energy, contributing to the multifunctionality and efficiency of solar panels, nanocomposites and nanocoatings are of relevance either in the photoactive layers, in protective layers, or on the surface of the solar panels. Energy harvesting by fullerenes have already been reported in the organic photovoltaic (OPV) industry [29]. As for packaging, for the outer layer, the use of nanostructured materials has been found to improve humidity, gas and UV barrier properties of solar cells in general. Nanotexturized surfaces and nanocoatings have been developed to provide self-cleaning effects to solar panels [30], therefore minimizing the requirements for maintenance while maximizing the energy yield.

In the subsequent sections, we review the different processing technologies that are used for nanocomposites conversion (including wet chemical and thermoplastic processing), as well as the common and new nanodeposition approaches with a specific focus on electrospray. The limitations and required process improvements are then discussed showing the challenges in nanoparticles dispersion.

In a following section, the different properties that can be enhanced using nanocomposites are reviewed, including barrier, mechanical, electrical/electronic, microwave absorbing properties and flammability resistance. Processability and compatibilization issues are also commented. The surface properties resulting from nanocoatings are then reported. The polymer nanocomposites applications are covered with a main focus on packaging, solar panels, and automotive sectors as well as a few words on other applications of interest.

Finally, the review gives some insights on safety and regulatory aspects that would influence the market uptake for new nanocomposites.

## 2. Processing

### 2.1. Nanocomposites

Several techniques have been developed for the preparation of polymer nanocomposites. The most important techniques are:

- Intercalation of the polymer
- In-situ intercalative polymerization
- Melt intercalation
- Direct mixture of polymer and particulates
- Template synthesis
- In-situ polymerization
- Sol-gel process

**Figure 1:**
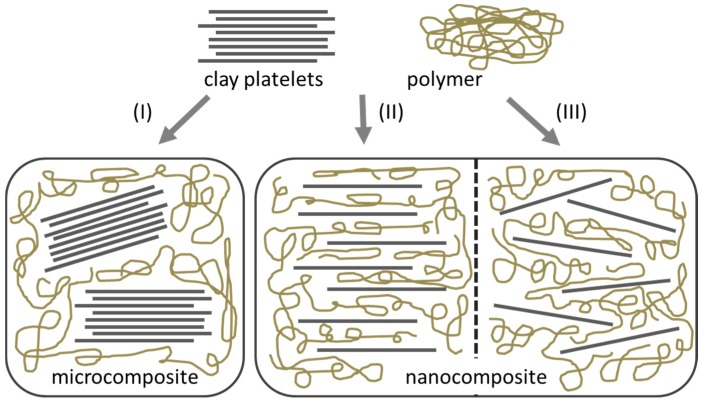
Different types of composites arising from the interaction of layered silicates and polymer. (I) Phase separated microcomposite, (II) intercalated nanocomposites and (III) exfoliated nanocomposites. Adapted from [31].

Intercalation processes is used for preparation of polymer-based nanocomposites, which contain layered silicates, as shown in Figure 1. In this method, a solvent is used in which the polymer or pre-polymer is soluble and the silicate layers are swellable. Nanocomposites prepared with this method have structures ranging from intercalated to exfoliated, depending on the degree of penetration of the polymer chains into the silicate galleries. Hence, this has become a standard method for the preparation of polymer-layered silicate nanocomposites [31,32].

When the polymer is unable to intercalate between the silicate sheets, a phase-separated composite is obtained, which have the same range of properties as that of traditional microcomposites (Figure 1). On the other hand, when polymer matrix enters between the layered silicates then intercalated nanocomposites structure occurs in a crystallographically regular fashion, regardless of the clay to polymer ratio. A well-ordered multilayer morphology built up with alternating polymeric and inorganic layers is generated. Normally, only a few molecular layers of polymer can be intercalated in these materials [31].

**Figure 2:**
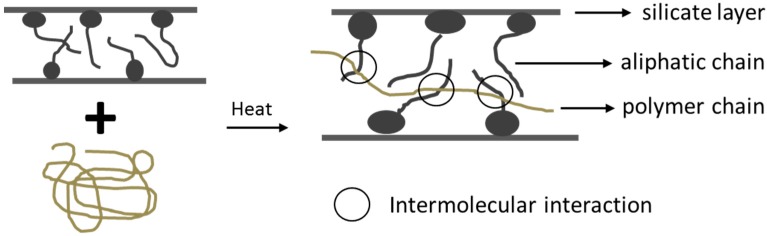
Melt intercalation synthesis of polymer/clay nanocomposite. Adapted from [27].

In-situ polymerization method encasing the layered silicate within the monomer solution, polymer formation starts between intercalated layered sheets by heat or radiation, suitable initiator or catalyst [15,16]. In the melt intercalation method, a mixture of the polymer and layered host are annealed above the softening point of the polymers statistically or under the shear. Diffusion of polymer chains from bulk polymer melt into the galleries between the host layers during the annealing process (Figure 2) [33,34].

In the template method, a template is used to form nanocomposite materials of particular shape for example layered, hexagonal shape, etc. The soluble polymer acts as a template for the formation of layers. This method is widely used for the synthesis of mesoporous materials but less developed for the formation of layered silicates [35,36]. In the direct mixing method, polymer or monomers are directly mixed with reinforcing phase [37]. In the in-situ polymerization method inorganic particles are dispersed into a precursor of the polymer matrix (monomer) and then polymerization of the mixture is done by addition of appropriate catalyst. In this method, simultaneous formation of nano particle and polymerization process occur [38,39].

Carbon-nanotubes (CNTs)-reinforced polymer nanocomposite materials are generally prepared by different methods, including direct mixing, solution mixing, melt-mixing and in-situ polymerization. Similarly, different processing techniques, mostly chemical and electrochemical methods, have been employed for the preparation of conducting polymer nanocomposites [40].

Despite the successful use of these different methods for the preparation of polymer-based nanocomposites, information on various factors is still lacking, such as the use of an appropriate method for a specific matrix reinforcement combination or the maximum amount of reinforcements to give optimum property combinations and lower the cost of the processes, etc. Therefore, it is still necessary to look into these aspects including the use of simulation and modelling techniques.

The most important requirements for a polymer based compound reinforced by nanoparticles are the combination of an optimum surface tension (caused by a good compatibility/interaction of the particle surface with the matrix) with a maximum dispersion of the separated/exfoliated particles. 

The physical requirements to achieve it are:

- similar to equal surface energy of polymer and particle surface
- low agglomeration energy
- low polymer viscosity
- high mixing efficiency in the process.

Due to the relatively high viscosity of polymers even at high temperatures, the best results can be generated by pre-dispersing surface-treated, i.e., organomodified particles in higher concentration (20–50 wt %) before “diluting” this masterbatch in the viscous matrix using a twin screw extruder with high throughput. 

Based on these requirements and the cost-performance ratio, there are three different ways for the production of nanoparticle-based composite concentrates to achieve an optimum, i.e., homogeneous, pre-dispersion and exfoliation of the particles/platelets in a thermoplastic polymer matrix:

**In-Situ:** The monomer is introduced between particle agglomerates or clay platelets by previous homogeneous mixing of the particles with the monomer or swelling of the agglomerates in the monomer and then polymerizing the monomers in between the particles (high solid content).

**Direct:** Polymerization and incorporation between nanoparticle agglomerates are performed together simultaneously. The polymerization is started after premixing the components by using temperature to initiate polymerization (e.g., radical initiators) or by a catalyst.

**Co-Precipitation:** Nanoparticles and low viscous solutions of the polymer are mixed together to form a homogeneous solution, then the finely dispersed polymer plus nanoparticle mixture is co-precipitated by adding another non-miscible solvent or by evaporating the solvent.

#### 2.1.1. Wet Chemical Processing

In wet processing techniques, solutions or suspensions are used for the formation of thin layer films, resulting in either stand-alone films (casting) or coatings on different substrates. When polymer based coatings are applied to a substrate via lacquering or spraying, which are the mainly applied techniques, the rheological properties of the coating formulation are decisive [41]. Drying techniques vary from ambient conditions drying to conventional hot air drying, infrared to microwave energy drying, while each method influences the film or coating properties [42]. Compared to extruded coatings, the applicable coating weight can be much lower, maintaining the desired barrier properties [43]. 

The use of nanoparticles in coating dispersions can bring many advantages to the resulting coating properties. Compared to multilayer films, nanocoatings demand lower material usage [27], being both an economic as well as ecological advantage. Additionally, surface coatings can be used for modulating repellent properties on various surfaces, e.g., for water repellent paper-based packaging [27] or easy-to-empty features [28]. For paper coatings, the most used nanomaterials are nanoclays, inorganic pigments, minerals, ceramics and starch [43].

#### 2.1.2. Thermoplastic Processing

**Figure 3:**
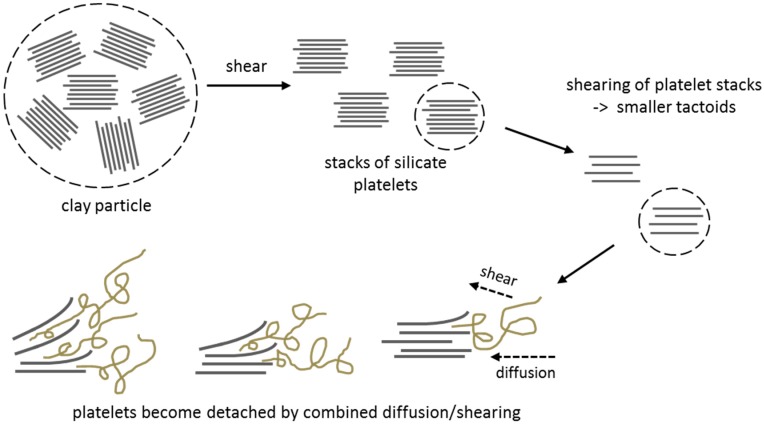
Mechanism of clay dispersion and exfoliation during melt processing. Adapted from [48,50].

Thermoplastic “dry” processing of polymers is mainly performed via extrusion, one of the most important polymer processing techniques. Extrusion allows melting a polymer with a high energy input during short time. Due to the supply of heat and energy input caused by friction between the screws, the mass melts, becomes formable and is pressed through the extruder die [44]. During the whole process, the mass can be compressed, mixed, plasticized, homogenized, chemically transformed, degasificated or gasificated [45,46]. When incorporating nanoparticles into polymeric compounds, different types of nanocomposites are possible. When processing the mostly desired exfoliated nanocomposites, the dispersion quality mainly depends on the extruder and screw configuration [47]. Exfoliation is favoured at high shear rates [9], while longer residence time favours a better dispersion [47]. Also, the location where the nanoclay is introduced has been shown to be an important factor [48]. However, the major factor whether a good dispersion or exfoliation is possible is the thermodynamic affinity between the nanoclay/nanoparticle and the polymer matrix [8]. When attractive interactions between the matrix and nanoclays are not sufficient, intercalation is reached, while exfoliation can be obtained when strong attractive interactions are present [49]. Figure 3 shows how exfoliation can be achieved via extrusion/melt processing [8]. 

The most widely used melt processes especially in packaging and automotive fields are injection moulding, film extrusion and extrusion coating. Since many different process parameters have a direct influence on the processed materials, Taguchi methods are commonly used in plastic injection moulding industry as a robust optimization technique for applications from product design to mould design; and from optimal material selection to processing parameter optimization. Villmow et al. [51] studied the influence of injection moulding parameters on the electrical resistivity of nanocomposites formed by polypropylene (PP) and carbon nanotubes (CNT) using a four-factor factorial design maintaining pressure, injection velocity, mould temperature and melt temperature. Sample with lower melt temperature and higher injection velocity shown a better dispersion compared with those injection moulded at low velocity and high melt temperature [51]. Chandra et al. [52] summarized their research on polycarbonate (PC) and CNT nanocomposite in order to achieve homogeneous distribution of CNT and to obtain high electrical conductivity the nanocomposites should be processed at high melt temperatures and low injection speeds to ensure proper and uniform electrical conductivity [52]. Recently, the F. Stan group has undertaken a study about the influence of the process parameters in the nanocomposite (PP/CNT) to improve the mechanical properties. The injection moulding parameters affect the degree of crystalline morphology of the moulded polymers. Therefore, these effects could affect the physical and mechanical properties of the injection moulded parts. On the other hand, the effect of crystallinity on the mechanical properties is less significant than the effect of the CNT. Their research work concluded that the most significant injection moulding parameter is the injection pressure [53].

Additionally, the use of compatibilizers can change the optimal parameters for the process. Constantino et al. studied the microstructure of the same nanocomposites PP/nanoclay produced by a non-conventional method of extrusion, SCORIM (Shear Controlled Orientation in Injection Moulding). This method is based on the concept of in-mould shear manipulation of the melt during the polymer solidification phase. The degree of clay exfoliation not only depends on the affinity and compatibility of the organoclay with the matrix, but also on the shear stress which is an extrinsic factor dependent on processing conditions and clay loading. High shear rate induced a thicker skin, while high temperature induced a thinner skin [18]. An interesting work was made by P.F. Rios, comparing the behaviour of different polymers with the same nanofiller. He studied the influence of injection moulding parameters in high-density polyethylene (HDPE), polyamide 6 (PA6), polyamide 66 (PA66), polybutylenterephthalat (PBT) and polycarbonate (PC) with carbon nanotubes. The main objective was to evaluate the electrical resistivity, thermal conductivity and the mechanical properties. The literature reveals how the different parameters of the injection moulding process might directly affect the quality of the injected part and their properties. The formulation is important, but the process parameters can be shown to have significant role too [54].

Film extrusion applications (mono- and multilayer) are found in food packaging sector, as coating substrates, ostomy films, etc. Typically, the coextrusion technology is used to obtain a multilayer film formed by different materials or compositions [55]. An example is found in the food packaging sector, the nanoreinforcement could be clays, silicates, cellulose-based, carbon nanotubes, graphene, starch nanocrystals, chitin/chitosan nanoparticles, silica nanoparticles (SiO_2_), and others. Each one has a function into the nanocomposite film, such as antimicrobials, among which the most common for food packaging are based on silver; O_2_ scavengers, nanosensors (reactive nanoparticles), etc. [56]. As discussed above, each type of nanofiller can influence the optimum process parameters. All systems should be optimized to obtain a homogenous material, and in the case of multilayer film, it is necessary consider the influence of the position, a number of layers and their thickness. When it comes to improving the productivity of the line, it is important to have an adequate measurement system and control, coupled to a head rapid response [57].

Extrusion coating is commonly applied on foil, paper, or fabric with polyethylene (PE), by extruding a web directly into the nip of a pair of rolls through which the substrate is passing. The high temperature is necessary to promote surface oxidation of the resin and to ensure adequate adhesion to the substrate [58]. The use of nanoparticles in extrusion coatings can generate or enhance properties like water vapour barrier, mechanical or heat sealing. 

### 2.2. Nanodeposits

Nanostructured coatings or nanocoatings consist in the covering of materials with a layer of materials at the nanometre scale according to the definition mentioned above, or covering of a nanoscale entity, to form nanocomposites and nanostructured materials using conventional or novel procedures such as vapour deposition, plasma-assisted/ion-beam-assisted techniques, chemical reduction, pulsed laser deposition, mechanical milling, magnetron sputtering, self-assembly, layer-by-layer coating, dip coating, sol-gel coating, TUFT (tubes by fibre templating) process, electrochemical deposition, sol-gel techniques or electro-hydrodynamic processing (comprising electrospraying and electrospinning techniques) [23,59]. Nanocoatings have been demonstrated to be highly applicable in the functionalisation of surfaces to provide specific properties such as antimicrobial [19], self-healing [20], flame retardant [21], gas barrier [22], etc. Much research has already been carried out in the biomedicine [60,61,62,63], sensors and electronics [64,65], textiles [23], solar panels [24], lithium-ion batteries [66], construction materials [67,68,69], packaging [22] and automotive fields [25], among others.

#### 2.2.1. Common Processing

The most common technologies for the roll-to-roll deposition of thin layers belong to the class of gas phase processes. These processes allow to coat flexible substrates with nano-scale inorganic or polymeric layers with thicknesses in the nanometre region up to some hundreds of nanometres [70].

Depending on the deposition mechanisms, gas phase processes can be classified as physical vapour deposition (PVD) or chemical vapour deposition (CVD). PVD processes are performed in a high vacuum and are based on the transfer of the solid coating material into the gas or vapour phase followed by the condensation on top of the substrate [71]. Typical materials to be deposited are metals and metalloids as well as their oxides, nitrides and carbides. The deposition of compounds is possible by reactive processes which are based on a chemical reaction between the material and process gases like O_2_, N_2_ or hydrocarbons, respectively.

The gaseous phase of the coating material is obtained either by evaporation due to electrical resistance heating or electron beam irradiation or by sputtering [71]. In the latter process, atoms of the solid coating material are released due to the impact of atoms of the process gas which were ionized and accelerated by an electrical field. The quality and barrier properties of the deposited layer can be improved by a magnetic field application during the process (magnetron sputtering) [72]. The fabrication of thin metal films on polymeric surfaces via PVD are commonly used techniques for the class of metal-polymer nanocomposites [73]. 

CVD processes are based on chemical reactions of gaseous precursors on the substrate surface [71,72]. The reactions are activated either by heat or by a plasma (PECVD = plasma enhanced CVD). These processes allow the deposition of inorganic or polymeric materials, e.g., SiO*_x_*C*_y_*H*_z_* [72], graphene [74] and parylene [75].

ALD (atomic layer deposition) is a modified version of a CVD process [71,76]. It allows the deposition of nearly defect free barrier layers which are well suited for the encapsulation of organic electronic devices.

**Figure 4:**
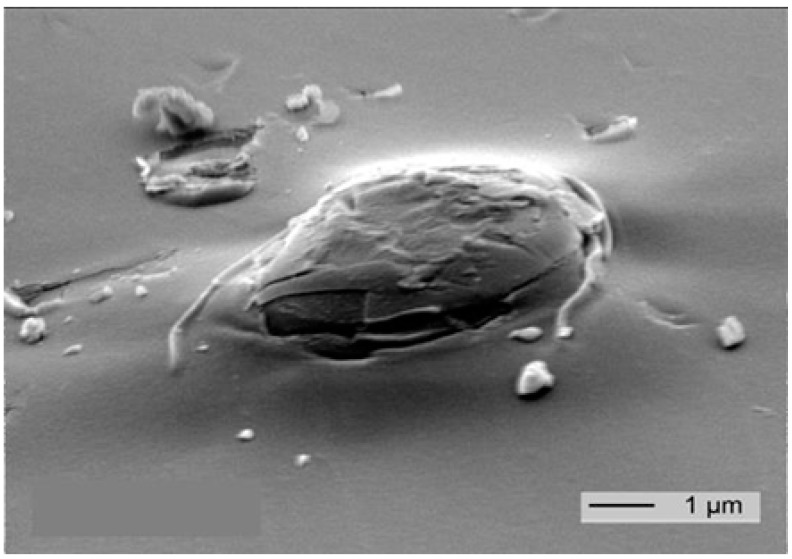
Surface of a polyethylene terephthalate film with particle, partially covered by a SiO*_x_* layer (Source: Fraunhofer IVV) [80].

The largest amount of layers produced by PVD or CVD processes on flexible substrates is used in food packaging technology [71]. The layers significantly reduce the permeation of oxygen and water vapour through packaging films and therefore increase the shelf life of packaged products. However, vacuum deposited layers are not completely tight since they contain defects formed during the deposition process (Figure 4) or due to mechanical stress [70,71]. The protection of vacuum insulation panels, flexible photovoltaic modules and organic light emitting diodes (OLEDs) against environmental influences therefore requires the combination of these layers with polymeric materials in complex multilayer structures exhibiting a high barrier performance [77,78,79].

Further applications of vacuum processing of polymer films cover the deposition of electrical conducting layers as transparent electrodes or to obtain antistatic properties as well as the modification of their optical properties [71]. 

#### 2.2.2. Electrospraying

Electro-hydrodynamic processing is a micro and nanofabrication technology comprising electrospinning and electrospraying [81,82]. Electrospinning is a process that produces continuous polymer fibres with diameters generally in the submicrometer range through the action of an external high-voltage electric field imposed on a polymer solution or melt. The electrospun nanostructures morphology is affected by the solution properties (mainly by the viscosity, surface tension and conductivity of the polymer solution) and by the process parameters (voltage, flow rate of the solution, tip-to-collector distance). Under certain conditions, capsules in the micro and nanoscale range can be obtained by adjusting the solution properties and the process parameters (e.g., lowering the polymer concentration and/or increasing the tip-to-collector distance), this particular process is generally known as electro-hydrodynamic atomization or electrospraying. Thus, electrospraying is a cost-effective technique that uses a uniform electro-hydrodynamic force to break up the liquids into fine particles. The injector is usually made in the form of a metal capillary, which is biased by a high voltage. When the electric repulsion in the solution or suspension exceeds the liquid surface tension a jet of droplets is produced [83,84,85].

The electro-hydrodynamic processing (EHDP) techniques are of particular interest as an alternative to conventional deposition and coating techniques since the latter ones require a controlled pressure and temperature environments. Compared to other deposition techniques, electrospray deposition (ESD) offers the advantage of a high deposition efficiency (up to 80%) and a reduction of the process steps [84]. The EHDP has been applied in several fields, mostly at a lab or a pilot scale. Although there have been some industrial efforts to scale up the electrospinning process, it has only been more recently that both techniques, i.e., electrospinning and electrospraying, have been scaled up via multinozzle injectors to an industrial level through companies such as Bioinicia S.L. 

### 2.3. Limitations and Process Improvements

One of the major problems when processing nanocomposites is the aggregation of nanoparticles, which is followed by an insufficient dispersion in the desired formulations. The agglomeration and aggregation takes place due to specific surface area and volume effects [86]. To characterize the dispersion quality, there are different techniques used for structure characterization of nanocomposites, naming X-ray diffraction (XRD), Scanning electron microscopy (SEM), Transmission electron microscopy (TEM), Infrared spectroscopy (IR) or Atomic force microscopy (AFM) [12]. For nanoparticles, TEM is the preferred method to examine the dispersion since polymer structure, void size and shape, filler size, shape and distribution, local crystallinity as well as crystal size can be determined. The degree of intercalation or exfoliation for particles presenting a specific layered organization such as clays can be characterized by XRD [26] and small angle neutron scattering can be used to characterize the fractal organization of amorphous particles like fumed silica [87]. Finally, recent efforts have been reported to monitor nanoparticles dispersion and nanocoating thickness distribution in-line during their processing [88].

#### 2.3.1. Dispersion Quality and Reaggregation

##### Particle Surface Modification

Most of the inorganic minerals have hydrophilic surfaces and are therefore not compatible with mainly hydrophobic polymer matrices. The aim of fillers surface modification is consequently their hydrophobization to enhance their compatibility with the polymer in order to enable intercalation or exfoliation. The resulting organophilic and hydrophobic clays, the layers of which have lower surface energy, contribute to polymer diffusion between the layers and finally clay platelets delamination [89]. This introduction of organic coatings can be carried out using physical and chemical interactions between filler and modifier.

Physical methods are performed either with low molecular weight surfactants or high molecular weight polymers. Surfactants generally include at least one polar group and an aliphatic chain. The modification is based on preferential absorption of the polar group to the high energy surface of the filler particles [90]. This exchange of inorganic surface cations with organic cations has been widely used for surface modifications of inorganic clays and conventionally realized with long-chain alkyl ammonium salts [89]. Another characteristic example is the treatment of calcium carbonate with stearic acid, forming a basic salt and decreasing the fillers surface tension and thereby influencing the composite properties [91]. The other technique displays a filler encapsulation with preformed or in-situ-formed polymers, developed by solution or emulsion polymerization, respectively [90].

To avoid modifier desorption from the particle surface, covalent bonds displaying chemical techniques can be used to improve interfacial interactions between the filler and matrix. Chemical surface modifications are mainly achieved via coupling agent treatments such as silane [86], titanate and zirconate to improve the adhesion between the polymer and particles, although here less information is available for nanoparticles than for microfillers [90]. Chemical grafting of macromolecules onto inorganic particles can be achieved through covalent bonding with the hydroxyl groups on the unmodified particle surface [86,90]. Compared to surfactants and coupling agents, this technique shows several advantages due to a wider selection of grafting monomers, enabling a tailor made nanocomposite structuring [90].

Beside conventionally used modifications with alkyl ammonium ions (mainly used for clays), advanced surface modifications of the fillers include modifications with reactive groups, initiator molecules or monomer molecules and several others approaches [89].

##### Ultrasonic Oscillations

Additional improvement of nanocomposite dispersion quality can be achieved via ultrasonic treatment. Ultrasonic cavitation transfers high amounts of energy, being able to disrupt physical and chemical interactions. Therefore, it has been widely used for dispersing, emulsifying, crushing and activating particles [17]. Additionally, ultrasound energy is able to break C–C bonds, leading to long-chain radicals formation. Those radicals might build chemical bonds on the clay surface in nanocomposite systems [49], and therewith enhance the polymer/filler compatibility but also that of immiscible polymers in general [92]. There are several studies on the use of ultrasonic oscillations for nanocomposite preparation [17,49,93,94]. Isayev et al. used high power ultrasound to break up silica agglomerates in ethylene propylene diene monomer rubber (EPDM), significantly reducing the agglomerate size [94]. Xia et al. ascertained reduced aggregation of nanosilica particles and also a redispersion in aqueous systems under ultrasound [17]. Lapshin et al. developed an ultrasound aided extrusion process for polyolefin-clay nanocomposites using MMT clays modified with quaternary ammonium salts in different polyolefin matrices. They detected increased basal spacing and even individual dispersed clay layers after ultrasonic treatment proved by TEM images [49]. 

##### Mechanical Alloying

This technique generally influences the particle size and shape for the synthesis of nanoparticles to prevent excessive aggregation at the following preparation of nanocomposites. Surfactant-assisted mechanical milling and alloying lowers the tendency to agglomerate by giving a steric barrier and also lowering the surface tension [95]. The milling process for crystalline nanoparticles with high energy ball milling can be divided into three stages. First, the particles undergo deformation localization in the shear bands, followed by a grain structure in nano-dimensions. The third stage is characterized by a random orientation of the grain and a peel off of single crystal nanoparticles [96]. With the aid of surfactants, hydrophobic surfaces can be obtained during the process prevent the newly formed fine particles from aggregation and determine size and shape of the final product, depending on the surfactant, grinding material and process, respectively [95]. Nandhini et al. produced nanocarbon with sizes ranging from 80 to 500 nm from graphite using sodium dodecyl sulphate/sodium dodecyl benzene sulfonate as surfactant in a high energy ball milling process [97]. Calcium carbonate particles of about 40 nm size could be obtained from a one-step grinding process, showing enhanced dispersion and grinding of particles when poly(acrylic acid, sodium salt) was used as surfactant [98]. Therefore, high energy ball milling can be applied for the production of various nanopowders that show good properties for the utilization in nanocomposites [99].

## 3. Material Properties

### 3.1. Nanocomposites

The development of polymer nanocomposites is currently one of the most active areas in the field of nano-enabled materials. As discussed, the simplest process strategy is just adding appropriate nanoparticles to a polymer matrix to enhance its performance often dramatically by simply capitalizing on the nature and properties of the nanoscaled filler. This strategy is particularly effective in yielding high performance composites [32,100,101,102,103,104,105,106,107,108,109,110,111], when good dispersion of the filler is achieved then the properties of the nanoscale fillers are substantially better than those of the matrix. That is why mainly very hard and stiff materials (minerals: oxides and silicates, carbon nanotubes) are used to optimize the main disadvantages of standard polymers [112] compared with the main competitive metal and glass materials: low modulus, insufficient creep resistance, low hardness/low scratch resistance, insufficient barrier properties, high flammability-low temperature resistance.

Nanofillers can significantly improve or adjust most of the different properties of the polymer base materials in which they are incorporated, sometimes also in synergy with conventional fillers and/or additives. 

#### 3.1.1. Barrier Properties

**Figure 5:**
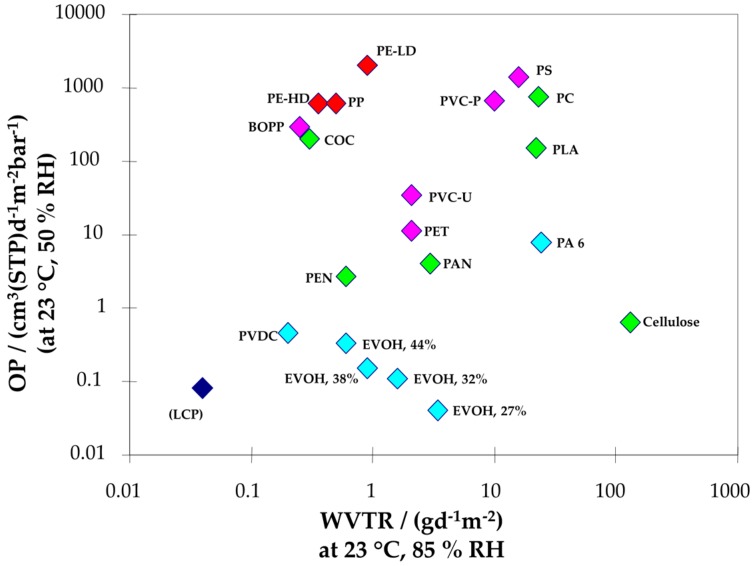
Comparison of oxygen permeability (OP) and water vapour transmission rate (WVTR) properties for different polymers normalized to 100 µm thickness. Source: Fraunhofer IVV [55].

Traditional packaging materials include metallic materials, ceramic (glass), cellulosic (paper and cardboard) and polymers. Due to the low weight, low cost, easy processability and the diversity of the chemical and physical properties of organic polymers, these are the most employed materials nowadays in the food packaging sector (around 40% of market share) [113]. The most frequently polymers employed for food packaging include polypropylene (PP), polyethylene terephthalate (PET), various types of polyethylene (LDPE, HDPE, etc.), polyvinyl chloride (PVC) and polystyrene (PS). Although polymeric materials have revolutionised the packaging sector and exhibit many advantages over traditional materials, their main disadvantage is their inherent permeability to gases and other small molecules. Figure 5 compares the permeability to oxygen and water vapour for different polymeric materials.

In general, the permeability of moisture or oxygen through polymers depends on various interrelated factors, which include:

- Structural characteristics and polarity of the polymeric chains
- Hydrogen bonding features and other intermolecular interactions
- Polydispersity and molecular weight
- Degree of cross-linking or branching
- Synthesis method and processing technology
- Crystallinity

The permeability to a specific molecule can be affected by the presence of others. For example, ethylene vinyl alcohol (EVOH), provides excellent barrier properties to oxygen in dry conditions. However, in very humid conditions (e.g., >75% relative humidity), its oxygen transmission rate can be increased by one order of magnitude, due to the swelling of the polymer by the presence of water molecules [114,115,116,117,118].

Because there is no pure polymer that exhibit all the required barrier and mechanical properties for every packaging application, polymer blends or complex multilayer systems are widely used. For example, to provide a high barrier to oxygen in a very humid environment, a material such as EVOH, which is sensitive to water, but very high barrier to oxygen, can be sandwiched between two layers of a hydrophobic polymer like PE [55,118,119]. Direct blending of polymers is also a used strategy to achieve desired barrier properties that cannot be obtained with monolayers of polymers [120,121]. Unfortunately, while polymer blending and multilayer films have afforded packaging materials with good barrier properties, these systems present high production costs, require the use of special adhesives that complicates their regulation, and are very difficult to recycle. Therefore, there still a great interest in the polymer industry to generate monolayer films with improved mechanical and barrier properties.

Polymeric nanocomposites are the latest materials aimed at solving the aforementioned problems [122]. Polymeric nanocomposites are prepared by dispersing nanoscale fillers throughout a polymeric matrix. In the literature, it many examples of polymeric nanocomposites containing as fillers layered materials such as clays, silicate nanoplatelets or graphene (Section 4.1.1), SiO_2_ nanoparticles [123], carbon nanotubes [124], starch nanocrystals [125], cellulose nanofibres and nanocrystals [126,127,128,129], chitosan nanoparticles [130,131] and other nanomaterials can be found. 

**Figure 6:**
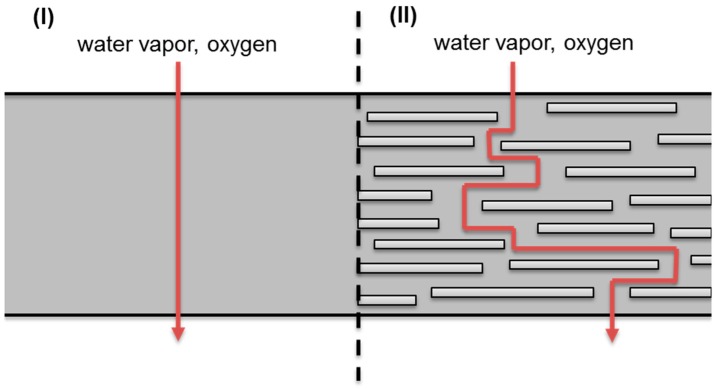
“Tortuous pathway” created by incorporation of exfoliated clay nanoplatelets into a polymer matrix film. In a film composed only of polymer (I), diffusing gas molecules on average migrate via a pathway that is perpendicular to the film orientation in a nanocomposite (II), diffusing molecules must navigate around impenetrable particles/platelets and through interfacial zones which have different permeability characteristics than those of the virgin polymer. Adapted from [133].

The dispersion of nanofillers into the polymer matrix affects the barrier properties of a homogeneous film in two ways. The first way is by the creation of a tortuous path for the diffusion of gas [132]. Due to the impermeable nature of the nanofillers, the molecules of gas must diffuse around them instead of taking a straight path perpendicular to the surface of the film. As a result, the mean path for the diffusion of the gas through the film is longer with the presence of nanofillers Figure 6 [133].

Taking into account this mechanism, it is evident than among all the different shapes of nanomaterials (spheres, fibres, rods, tube, plates), layered nanomaterials (2D) are the most appropriate ones to improve the barrier properties and therefore this type of materials is the most studied for this application (Section 4.1.1). Different models to predict barrier properties of nanocomposites depending on the filler geometry and the ratio have been proposed and are listed in Table 1.

Beside nanofiller content and aspect ratio, the state of exfoliation significantly affects barrier properties of nanocomposites [139]. Exfoliation levels are, however, not included and have to be taken into account when using such models.

A second way in which nanomaterials can influence the barrier properties is by causing changes in the polymeric matrix itself. If the interactions nanomaterial-polymer are favourable, the polymer chains in the proximity to the nanomaterials can be partially immobilized. Therefore, the gas molecules that migrate through these interfacial areas will have attenuated movement. The effect of the interfacial region is especially important in polymeric matrices that exhibit very high permeability to gases, such as polyolefins [140].

In any case, every nanomaterial-polymer system is different and the properties can only be predicted in general terms. The consideration mentioned above demonstrate why the nanomaterials have been successful as fillers for improving barrier properties of polymers. Compared to micrometric fillers, the nanoscale fillers have much higher aspect ratios and, due to this higher surface area to volume ratios, the interfacial volume in a nanocomposite film is much higher than that of a polymer microcomposite formulated from the same materials.

#### 3.1.2. Mechanical Properties: Reinforcement and Light Weighting vs. Conventional Composites

**Figure 7:**
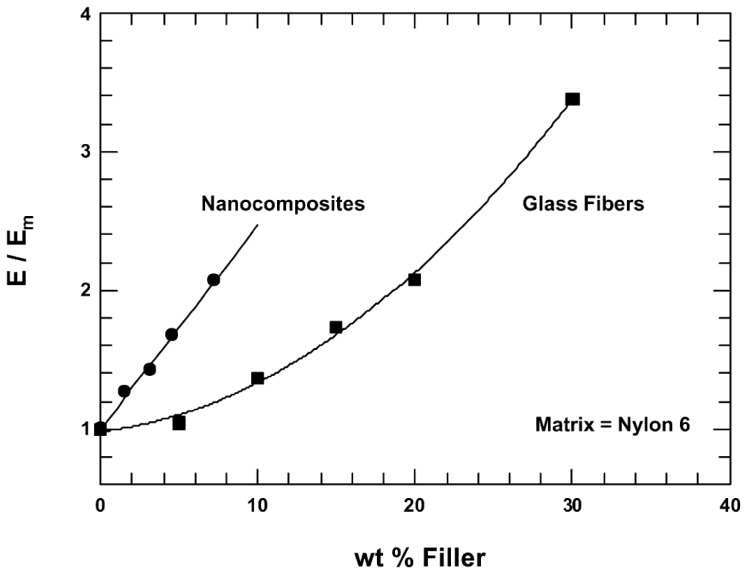
Comparison of modulus reinforcement (relative to matrix polymer) for nanocomposites based on montmorillonite (MMT) versus glass fibre for a PA 6 matrix [141].

Nano fillers are used in polymer matrix to improve mechanical properties such as stiffness, strength via reinforcement mechanism [141,142,143]. In case of fumed silica, different reinforcement effects were reported depending on the nanoparticles dispersion state, surface area, polydispersity, and organo-modification, possibly leading to their grafting to the matrix [144]. It is proved that properly dispersed and aligned clay platelets are very effective to improve stiffness of polymer matrix material. By comparing the increase in the Young’s modulus, *E*, of injection moulded composites based on nylon 6, relative to the modulus of the neat polyamide matrix, *E*_m_, when the filler is an organoclay versus glass fibres [141]. In this example (Figure 7), increasing the modulus by a factor two relative to that of neat nylon 6 requires approximately three times more mass of glass fibres than that of MMT platelets. Thus, the nanocomposite has a light weight advantage over the conventional glass fibre composite. Furthermore, if the platelets are aligned in the plane of the sample, the same reinforcement should be seen in all directions within the plane, whereas fibres reinforce only along a single axis in the direction of their alignment [143]. In addition, the surface finish of the nanocomposite is much better than that of the glass fibre composite owing to nanometre size of the clay platelets compared to the 10–15 µm diameter of the glass fibres.

The modification of the polymer matrix with clay seems to be a proper mean to increase the mechanical stability of the polymer melt. This effect is advantageous for some polymer processing techniques like blown film manufacturing or extrusion blow moulding. Due to their ability to act as a nucleating agent, clay particles may induce changes in the crystallinity (morphology and crystal type) of the matrix polymers like PA6 or PP [145,146].

Several characteristics of polymers can be influenced by the size and geometry of the nanoparticles, e.g., the Young’s and shear moduli [147,148,149,150,151,152,153], the thermal expansion coefficient [147,149,154], the thermal [155,156] and the electrical conductivity [157]. Furthermore, it was shown that, even when the polymer matrix evolves from the glassy state to a rubbery state, the influence of nano-reinforcement on the thermo-mechanical response is still observable [158,159,160,161]. It can be concluded, that polymer nanocomposites are favourable in many industrial domains such as aerospace, automobile, and electronic packaging, where they act as multifunctional structures.

The interface is an important characteristic for nanocomposites. Therefore, a lot of energy has been invested to describe the mechanical response and determine its effective stiffness and volume fraction (or thickness) [147,148,150,151,155,156,159,160,161,162,163,164,165,166,167,168,169,170,171,172]. However, up to now, problems in the experimental visualizations in nanoscale hindered from understanding the exact thickness and properties of the interphase region. Consequently, the dimension of interphase layer in nanocomposites was mostly analysed by indirect methods. For example, the glass transition temperature was determined for the continuous polymer and additionally for the interphase by DSC (differential scanning calorimetry) by Mortezaei et al. [170]. In comparison, thermally stimulated depolarization currents (TSDC) were used by Fragiadakis et al. [171]. Based on that, they calculated the dielectric strength of the corresponding relaxation. This they connected with the degree of crystallinity of the bulk polymer and then calculated the interfacial region. AFM (atomic force microscopy) was used by Bhuiyan et al. [164,165] in order to estimate the morphological changes of the regions around the particle surface.

Apart from measurements, mechanical design and modelling have been used to understand and investigate the reinforcing effect of the intermediate medium between nanoparticles and the surrounding polymer. Therefore, nanocomposites have been considered as a three phase multi-inclusion using a continuum model. The third phase is the densified polymer region of the nanoparticle, which is represented as an independent and different material. Consequently, it shall be possible to characterize the material first by experiments. In the next step, the constitutive equation of the micromechanics model or the numerical solution of the finite element shall be fed with information about the particles and the homogeneous phase. Information about the third phase—the interphase—can then be extracted by solving the equations inversely. 

As mentioned before, DSC can be used for evaluating nanocomposites. However, the degree of crystallinity and crystallization rate can be affected by crystallization in narrow spaces. If the space is so narrow, that the spherulitic growth is restricted, primary nuclei are not available for heterogeneous crystallization. Consequently, homogeneous nucleation appears. This can lead to a low crystallization rate, degree of crystallinity and melting point. This was witnessed in phase separated block copolymers [173,174] and polymer blends [175]. Moreover, restricted crystallization of linear polyethylene in nanoporous alumina led to homogeneous nucleation for pore radii of 31–55 nm but heterogeneous nucleation for 7.5–24 nm pores [176]. Syndiotactic polystyrene [177] as well as linear polyethylene [178] showed decreased crystallinity versus bulk crystallization in nanoporous alumina. By adding nano-particle incorporation in a polymer, parallels to confined crystallinity, nucleation effects and disruption of attainable spherulite size appear.

With the incorporation of inorganic and nano particles, nucleation of crystallization can appear. At nano scale, the nanoparticle can replace for the lack of primary nuclei consequently rivalling with the confined crystallization. At higher nanoparticle concentration, lower crystallization kinects can be obtained due to the higher viscosity (lowered chain diffusion rate). Here it becomes obvious, that the crystallization process underlies multiple factors and is affected by many causes. At low concentrations, nucleation of crystallization was observed by the onset temperature of crystallization (*T*_c_) and crystallization half-time. This was visible in several composites, such as poly-(3-capro-lactone)-nanoclay [179], polyamide 66-nanoclay [180,181], polylactide-nanoclay [182], polyamide 6-nanoclay [183], polyamide 6.6-multi-walled carbon nanotube [184], polyester-nanoclay [185], poly(butyleneterephthalate)-nanoclay [186], polypropylene-nanoclay (sepiolite) [187] and polypropylene-multi-walled carbon nanotube [188]. At higher concentrations, rather a delay of the crystallization rate has been monitored. This was even seen in such composites, where nucleation appeared at low concentrations [180,184,186,188,189,190]. The reason for the retardation of crystallization at higher concentration can be reasoned with diffusion constraints.

Similar effects were observed in a trial, where unmodified and organically modified clay were mixed in maleic anhydride grafted polypropylene [191]. The unmodified clay led to nucleation. In comparison, exfoliated clay led to a lower crystallization rate. This was also explained in [191]. While nucleation appears in many composites, the crystallization rate is commonly lowered predominantly at higher concentrations. 

As mentioned before, changes in nano composites can be monitored via the glass transition temperature *T*_g_ before and after adding nano particles. However, not only increases but also decreases were observed. The reason for this is the different interactions between polymer and particle. 

The *T*_g_ of a polymer is influenced when the chain is surrounded from another phase for several nanometres. An extreme case of this is where the other phase is air (or vacuum). Then the glass transition temperature of the polymer at the interphase or also in thin films (<100 nm) can be minor compared to the *T*_g_ in the bulk material [192]. This can also be reflected as a confinement effect. A specific experimental example was reported where poly(2-vinyl pyridine) showed an increase in *T*_g_, poly(methyl methacrylate) (PMMA) showed a decrease in *T*_g_ and polystyrene showed no change after the addition of silica nanospheres. These dissimilarities were related to surface wetting [193]. The *T*_g_ decrease for PMMA was explained by the free volume existing at the polymer interface due to poor wetting. In most publications only modest transformations are described (<10 °C) as noted in various examples tabulated in Table 2. In some cases, the organic modification of clay can result in a decrease in *T*_g_ due to plasticization [194]. It should be noted that the values noted in Table 2 involved relatively low levels of nanoparticle incorporation (<0.10 weight fraction and even lower volume fraction) and larger changes in *T*_g_ could be expected at much higher nanofillers volume fraction.

#### 3.1.3. Viscosity = Processability vs. Mechanical Properties

Above a certain value for the molar mass *M*_c_, the zero shear viscosity of polymer melts relates to the molar mass to the power of 3.4 [195,196]. The relation can be explained by a network due to entanglement, where macromolecules are crosslinked. This network can be described by an average molar mass which is embedded between two crosslinks. Although low viscosities, i.e., small molar masses, are favoured for processing, higher molar masses lead to better mechanical characteristics like toughness and strength. Introducing nano particles into the polymer might be a solution to fulfil both requirements: mechanical stability and simple processability. 

Nevertheless, many hurdles are yet to be overcome, especially regarding the dispersion and the processing of these materials. Mackay et al. [197,198] described a reduction of the viscosity of nanoparticle-filled polymer melts over a large concentration range. However, this does not follow the idea of Einstein, according to which the viscosity should increase with volume fraction and the viscosity of the polymer. Comparably, Merkel et al. [199] reasoned the reduction of the viscosity by the reduced free volume induced around the nanoparticles. This leads to a strong decline in the glass transition temperature. This may affect the eventual characteristics. Contradictorily, Kharchenko et al. [200] described a rising viscosity of carbon nanotube-filled polymer materials, even at low concentrations. Similar was reported in [201], where the goal was reached and processing as well as mechanical properties of PP were improved by the additivation with silica nanoparticles. Therefore, newly modified porous, semi-crystalline PP powders developed [202]. 

#### 3.1.4. Polymer Blend Compatibilization

For the compatibilization of polymer blends, two factors should be addressed firstly. This is the interfacial tension between the phases which should be reduced and the avoidance of coalescence of the nanoparticles. One solution to address this, is the additivation by graft or block copolymers. Its constituents should be equal to or as least compatible with the blend components. Observations have been reported, in which the incorporation of nanoparticles even prevented the coalescence after shear mixing. Examples can be found in [203,204,205,206,207]. 

One reason for such observations might be that the nanoparticles accumulate at the interface. This avoids coalescence by a barrier-type mechanism. Another explanation might be that nanoparticles behave like graft or bloc copolymers, which accumulate at the interface and are bound by physical [208] or chemical interactions [205]. 

#### 3.1.5. Flammability Resistance

More and more applications in the fields of buildings, transports or even aeronautics require enhanced fire retardant properties. Some car manufacturers also follow specific standards regarding flammability of car equipment [209]. It has become an area of increased research for sustainable alternatives due to toughening of safety standards, especially regarding halogenated compounds. Therefore, many studies are currently carried out in order to develop new environmentally friendly/halogen-free fire retardant additives or to increase their efficiency [210]. The target of fire retardant additives is to reduce heat released bellow the self-sustaining level of the fire. The mechanisms of action that can be involved in the enhancement of the fire resistance can be: (i) formation of a ceramic-like protective shield; (ii) decrease of heat transfer inside the material; (iii) creation of a physical barrier to oxygen propagation; (iv) modification of the degradation mechanism. The problem arising for most conventional fire retardant compounds is the large amount needed (typically up to 60 wt %), so that mechanical properties are affected. An increasing attention is paid to other routes such as addition of nano-fillers [211], allowing an enhancement of the fire resistance and of the mechanical properties at the same time. Several studies were realized about the effect of clays into various polymers showing a large increase of the fire resistance [212,213,214]. While nanofillers are often insufficient to meet standards for flame retardancy, the most promising approaches is often pointed to consist in the combination of standard flame retardant additives and nanofillers. The impact of dispersion on the flammability of a material is driven by the barrier to oxygen permeation that can prevent further feeding the combustion, as well as by a charring effect when nanoparticles form a cohesive surface protective layer to stop fire propagation [7]. Likewise, continuous nanoparticles coatings, e.g., flame retardant paints, could have similar effect.

#### 3.1.6. Electrical Properties—Electronics 

Polymer nanocomposite can show conductive properties for electronic and electrical applications. The electrical conductivity of carbon nanotubes in insulating polymers has also been an important topic of interest. The potential applications include transparent conductive coatings, supercapacitors, electromechanical actuators and various electrode applications [215,216]. Nano composite based polymer with various nanoscale filler inclusions have been investigated for sensor applications including gas sensors, biosensors and chemical sensors. The nanofillers employed include metal oxide nanowires, carbon nanotubes, nanoscale gold, silver, nickel, copper, platinum and palladium particles [217]. However, most metal-polymer nanocomposites using gold or silver nanoparticles, for example, are rather generated from a deposition process [73], yet still their electrical properties and possible scope of application stay the same. With carbon nanotubes, the electrical resistance was found to be significantly changed by exposure to specific gases such as nitrogen dioxide and ammonia [153]. A nanocomposite of single wall CNT/polypyrrole yielded gas sensor sensitivity similar to SWCNT alone [157]. The sensing capability of these nanocomposites can be based on conductivity changes due to gas or chemical interactions between nanofiller or the conjugated polymer [159].

In order to fulfil the potential applications of conducting polymer (CP) nanotubes and fibres, it is necessary to understand the electronic transport properties of individual polymer tubes/fibres. For template prepared nanofibres, the easiest and usual way is to leave the synthesized polymer fibres inside the pores of the template membrane and measure the bulk resistance across the filled membrane by a two-probe method. The measured membrane resistance can be used to estimate the conductivity of a single fibre. It was found that at room-temperature, conductivity of 30-nm polypyrrole and poly(3-methylthiophene) fibres are much higher than in conventional forms of the analogous polymer [160,161].

#### 3.1.7. Microwave Absorbing Property

Conducting polymer in the form of new microwave absorbing materials have been explored due to their lower density and their easy processability. In general, traditional films or pellets of doped polyaniline and polypyrrole exhibit an electrical loss in the microwave frequency (*f* = 1–18 GHz) [166,168]. This may arise from an enhanced chain ordering induced by the tubular morphology [169]. It was found that the doped polyaniline with fibre-like morphology has better electromagnetic wave absorbing property than that of polyaniline with particle-like morphology [170].

### 3.2. Nanodeposits

#### 3.2.1. Repellence to Selected Liquids

The use of repellent surfaces can be of great interest for many applications such as anti-fouling, self-cleaning, anti-smudge or low-drag for different industries. Hydrophobic surfaces are those capable of repelling water, oleophobic surfaces are capable of repelling oils. To achieve super hydrophobicity or superoleophobicity, the contact angle of water or oil, respectively, must be higher than 150° and rolling of or sliding angles should be small [218]. Superomniphobic, also called superamphiphobic, surfaces are those with contact angles greater than 150° and low contact angle hysteresis (generally below 10°) for liquids with low and high surface tension values [219]. Compared to a superhydrophobic surface, it is more difficult to create a superoleophobic or superamphiphobic surface because the surface tensions of organic liquids are appreciably lower than that of water [220]. Nevertheless, surface roughness, surface energy and the structure of the solid substrate should be adjusted to create superoleophobic as well as superhydrophobic surfaces and the strategy is similar for the creation of both.

Normally, superhydrophobic surfaces are obtained by mimicking those occurring naturally in plant leaves, like lotus or elephant ear [221], having an optimal combination of surface roughness and low surface energy. The surface roughness should have a hierarchical structure at the micro- and nano-scale, which for lotus leaves are built by convex cells and a much smaller super-imposed layer of hydrophobic three-dimensional wax tubules [222] and in the case of elephant ear arise from micro-bumps formed by convex surface papillae and low surface energy resulting from the formation of a crystalline wax film [223]. It is well known that those micro- and nano-scaled surface structures are of critical importance to the surface water repellence, so the use of nanoparticles is one easily accessible alternative to mimic the multilayered structures of the natural prototypes of non-wettable surfaces [224]. Different nanoparticle composites based on SiO_2_, TiO_2_, Al_2_O_3_, Fe_2_O_3_ and Au have been used. Among them, SiO_2_ nanoparticles are the most commonly used [225,226,227,228,229] due to their simple synthesis procedure, easy modification and compatibility with superhydrophobic surface coatings.

Self-cleaning surfaces can be achieved by two different approaches: creating a superhydrophilic surface or creating a superhydrophobic surface. In the first approach water completely covers the surface with a continuous film and washes away any dirt [230]. This can be achieved by incorporating photocatalytic chemicals as TiO_2_, with a contact angle with water below 1° and which under UV light generates activated oxygen that decompose the organic materials on the surface, having a dual effect. In the second case, over a superhydrophobic surface, the rolling droplets pick up dust particles, easily removing them from the surface, thus achieving the self-cleaning effect.

Synthetic hydrophobic coatings often employ heavily fluorinated species due to the high surface energy of these materials when in contact with water. In many cases superhydrophobic coatings are created by combining perfluoroalkyl substituents on a nanoparticle (generally SiO_2_), having significant flexibility through a range of particle sizes and surface functionalizations [231].

When preparing superhydrophobic coatings for solar panels (and also for windshields for automobiles, safety googles, etc.), high transparency is also needed. To achieve this goal, the coating must be composed of low-light absorbing materials with refractive indices spanning the refractive indices of air to that of the substrates [232]. Among the conventional transparent materials, silica has the lowest refractive index, absorbing minimal visible light. Li et al. repaired a superhydrophobic coating based on a nanoscale porous structure spontaneously assembled from branched silica nanoparticles [233].

Different methods and processes have been used for the production of super hydro/oleo/amphiphobic surfaces based on nanoparticles. The hierarchical structure in the micro- and nanoscale consists of more than one layer of protrusions on the surface, with the bigger particles at the bottom and the smaller particles on the surface. One alternative is the use of nanoparticle-assisted lithographic techniques, which rely on masks to create geometric patterns on surfaces, adjusting the roughness by changing the etching duration and the lattice space of nanoparticles with different sizes. Chemical [234] or reactive-ion etching [235] processes have been studied to remove the polymer matrix.

Layer by layer (LBL) deposition is a process of constructing multilayered films based on alternating the charge of a substrate, with no need of a special environmental chamber to control the reaction conditions unlike plasma treatment or chemical vapour deposition (CVD). To create the roughness structures for desired surface wettability nanoparticles are often added into the solutions. Cao and Gao [236] fabricated transparent superhydrophobic and highly oleophobic coatings through LBL assembly of 20 nm silica nanoparticles and sacrificial 60 nm polystyrene nanoparticles, that were removed afterwards by calcination.

Self-assembly processes, in which the interactions among the components in solutions spontaneously form an organized distribution, have also been used. Particularly, to organize nanoparticles in a molecular way, self-assembled monolayers (SAMs) designed to have a specific and favourable interaction with the solid substrate of interest are used [237]. Lassiaz et al. fabricated a stable hydrophobic surface by using the reaction of octylphosphonic acid with the surface of alumina nanoparticles [238].

Electro-hydrodynamic techniques (electrospinning and electrospraying) have also been used to create superhydro/oleo/amphiphobic surfaces, as is well described by Sas in a review of the topic [239]. Electrospinning can produce fibres with various diameters and a low fibre diameter introduces one degree of roughness to the electrospun materials. By tuning the electrospinning parameters, post-treatments steps or using some additives in the polymer solution, the second scale of roughness needed for superhydrophobicity is created. Polystyrene (PS) and their copolymers are the most frequently used due to their low surface energy, low cost and easiness of use for electrospinning. Nanoparticles like polytetrafluoroethylene [240], titania or graphene [241] can also be added to increase PS roughness. Other non-fluorinated [242] and fluorinated [243] polymers have also been used to fabricate superhydrophobic surfaces. Hydrophilic polymers can also be coated by annealed electrospun nanostructured fibres of hydrophobic silanes to achieve a hydrophobic surface and reduce moisture sensitivity [244]. However, the challenge of achieving such electrosprayed superhydrophobic and especially superamphiphobic surfaces with strong adhesion to the substrate and with non-intended migration, using application complying materials remains a challenge; this challenge is even greater when it comes to food packaging applications. 

Other non-conventional approaches have been used to create this type of surfaces. Deng et al. [245] created a super amphiphobic coating by depositing a soot layer onto a glass slide held above the flame of a candle. The resulting soot consisted of carbon particles with a typical diameter of 30 to 40 nm, forming a loose, fractal like network. This layer exhibited superamphiphobic behaviour but was extremely fragile. It had to be coated by a silica shell using CVD techniques. In a latter study, a paraffin wax was used to fix the candle soot, creating a paraffin wax-fixed candle soot (PFCS) coating [246]. This PFCS coating method has been tested on various surfaces, such as metal, ceramic, wood, plastic and paper.

Some of these processes and materials have already been patented. Huang developed a hydrophobic and lipophobic coating material comprising nanoparticles with a determined molecule for easy clean touch screens [247]. S. C. Johnson and Son, Inc. patented the process as well the composition of a coating for producing surfaces that are self-cleaning by water which contains an aqueous mixture of nanoparticles and surface modifier [248]. Cas Guangzhou Chemistry Co. Ltd. patented a super-hydrophobic or super-amphiphobic coating based on blending nanoparticles, epoxy resin with a solvent to obtain the epoxy resin hybridization solution [249]. Ashland Licensing and Intellectual Property Llc patented a coating composition and process for generating transparent, near-transparent, and semi-transparent super-hydrophobic coatings whose composition comprises hydrophobic nanoparticles of silsesquioxanes containing adhesion promoter and low surface energy groups [250].

Also, some technologies have been patented for producing hydro/lipo/amphi-phobic surfaces referring to packaging materials [251,252,253]. For example, Eka Nobel patented a paperboard packaging with hydrophobic zeolite that enhances their water-repellent capacity [254] and Bostik Findley Sa patented a system for gluing hydrophobic and oleophobic substrates that are intended for packaging [255].

#### 3.2.2. Self-Cleaning through Photocatalysis

To avoid the adherence of liquids, in addition to the lotus effect that nanocoatings can provide, they can also promote other self-cleaning mechanisms by, e.g., photocatalytic effect, which allows the chemical decomposition of many organic pollutants.

TiO_2_, the most thoroughly semiconductor investigated in the literature, seems to be the most promising compound for this purpose. As photocatalysis is an interfacial phenomenon, nanostructured TiO_2_ surfaces exhibit superior photocatalytic activity due to a high surface area-to-volume ratio. There are common ways of applying coating with photocatalytic TiO_2_. Among these various deposition systems, ESD (Electrostatic Dissipative Coating) is attractive because it produces extremely fine (sub-micron), selfdispersive (non-agglomerating), highly wettable (electrowetting), adhesive droplets that yield a uniform coating on the substrate. The pigmentary properties are no longer relevant for nanostructured particles, giving therefore almost transparent composite. TiO_2_ semiconductor provides the best compromise between catalytic performance and stability in aqueous media [256,257].

Among the different phases, TiO_2_ anatase is one of the most promising photocatalyst because of its high oxidative power, abundance and chemical stability. Under UV-illumination, it generates electron-hole pairs able to degradate organic matter or even microorganisms. Unfortunately, it can only absorb UV light (about 5% of solar light) which is a key drawback for its widespread application. As a consequence, efforts have been devoted to extending the light absorption of TiO_2_ to the visible region. Tudor et al. have developed nanostructured anatase (particle sizes < 20 nm) doped with Ag through hydrothermal process to obtain photocatalytic materials with sensitivity in the visible region of solar spectrum [258]. Further benefiting from its antimicrobial capacity, self-cleaning active textiles could be manufactured applying by electrospray this specifically doped nano-TiO_2_ on fabrics [259]. Another recently studied application using the photocatalytic effect of such nanocomposites is the incorporation of active TiO_2_ nanostrucures in poly (methyl methacrylate) (PMMA) for the removal of dyes, phenols and bacteria from water [260]. This approach benefits from the lack of need for a recovery of the active nanoparticles after water treatment due to their immobilization. 

## 4. Applications of Polymer Nanocomposites

### 4.1. Packaging

#### 4.1.1. Barrier Materials

As indicated above in Section 3.1.1, the most studied and promising materials used to improve the barrier properties of polymeric nanocomposites destined to packaging are layered nanomaterials. Two main types of layered nanomaterials have been studied as nanofillers to decrease the permeability to gases of polymers: layered silicates (such as nanoclays) and graphene based materials (graphene and graphene oxide). Nanoclays have been extensively investigated over the last decades because of their excellent barrier properties, low price and food contact compatibility [133]. The use of graphene in nanocomposites is much more recent, but several examples in literature have shown that it can be a strong candidate for gas-barrier materials [261]. A description of the most representative polymeric nanomaterials for packaging based on these two types of layered materials is shown in the following paragraphs.

##### Clay Nanocomposites

**Figure 8:**
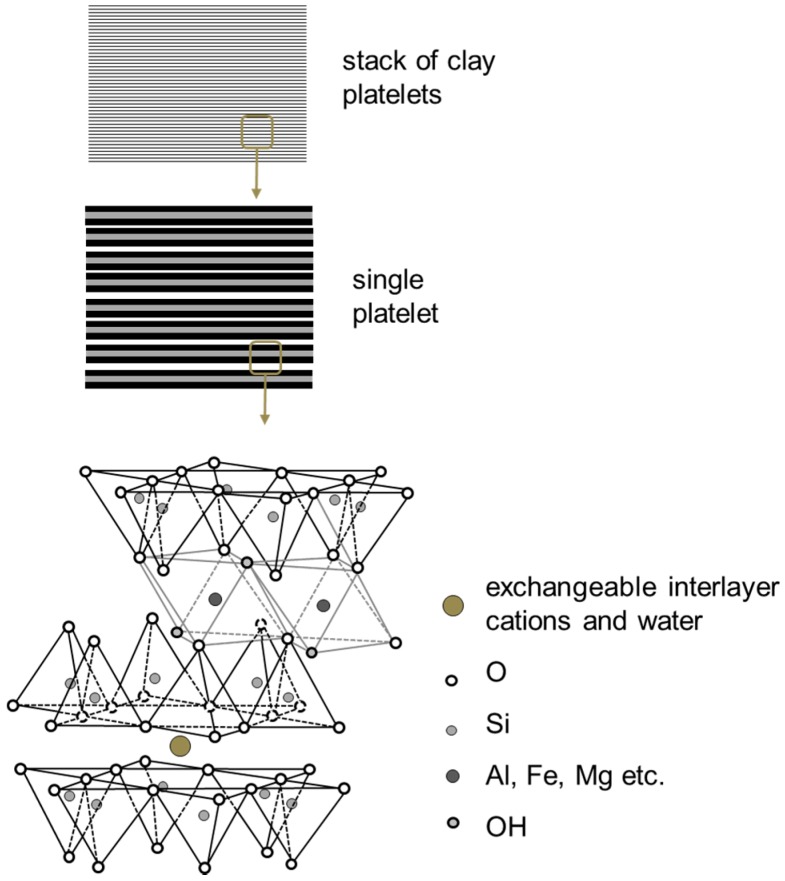
Structure of montmorillonite (phyllosilicate clay). Adapted from [133].

By far the most studied nanoscale fillers for polymeric nanocomposites destined to packaging are nanoplatelets composed of clays or other silicate materials. Nanoclays popularity’s for packaging application is due to their low cost, stability, effectiveness and benignity. Montmorillonite [(Na,Ca)_0.33_(Al,Mg)_2_(Si_4_O_10_)(OH)_2_·*n*H_2_O)], a layered phyllosilicate composed of anisotropic layers separated by water molecules, is the typical clay used for polymeric nanocomposites (Figure 8). The platelets have an average thickness of ~1 nm and lateral dimensions ranging from tens of nanometres up to several microns. Each platelet contains a layer of magnesium or aluminium hydroxide octahedra sandwiched between two layers of silicon oxide tetrahedra. Each face of the platelet has net negative charge, which is compensated by interlayer cations (Ca^2+^, Mg^2+^, Na^+^, etc.).

Individual layers of montmorillonite have surface areas in excess of 750 m^2^/g and aspect ratios in the order of 100–500 [26]. These structural characteristics contribute to the excellent utility of montmorillonite as nanofiller in composites, significantly increasing the mechanical and barrier properties of the polymer with a few percent addition into the matrix. Montmorillonite is not the only layered silicate used in polymeric nanocomposites. Related clays such as saponite, hectrite and kaolinite have also been used and have shown properties improvements [262].

During the last two decades, hundreds of polymer-clay nanocomposites have been described, and nanoclays have been incorporated into every important class of polymer, both synthetic and natural. Some representative examples of clay nanocomposites whose polymeric matrices are typically used in packaging are provided in Table 3, along with selected moisture and oxygen permeability data. This table shows just some examples that have been studied. A more comprehensive list can be found in the literature, consulting any of the numerous specific reviews on the subject [12,263].

##### Graphene Based Nanocomposites

Recently, graphene has received significant attention and has become one of the most studied materials due to its superior properties. Graphene, a monolayer of graphite, has not only excellent mechanical, electronic and optical properties [273], but also is considered an ultrathin, perfect two-dimensional (2D) barrier against gas diffusion [274], since the electron density of aromatic rings in graphene is high enough to repel the penetration of atoms or molecules [275].

When compared with clays, graphene nanoplatelets can have some advantages as two-dimensional nanofillers for polymer nanocomposites. Polymers incorporating graphene show not only enhanced gas barrier properties but also reinforced mechanical strength and improved thermal properties and electrical conductivity when properly dispersed in the polymer matrix [276]. As compared with other nanocarbon forms, such as fullerenes or carbon nanotubes, graphene has a higher surface-to-volume ratio (aspect ratio) and so will be able to achieve the longest gas-diffusion pathway, even at low loadings. The main drawback when using this promising material is that the synthesis of defect-free, large-area, monocrystalline graphene at large scale is still challenging and too expensive for packaging application [277]. One alternative for the use of the gas-barrier properties of graphene in mass production is to use graphene oxide (GO) or its reduced form, reduced graphene oxide (rGO). GO, which consist of oxygen-containing functional groups on the basal plane [278], can be produced at large scale in polar solvents and can be well-dispersed with high aspect ratio in hydrophilic polymers [279].

Some representative examples of graphene-based nanocomposites targeting to improve gas barrier properties for food packaging are presented in Table 4. Polymer nanocomposites are mainly produced by solution mixing and melt processing.

For graphene-nanocomposites the platelet size, stacking orientation and degree of graphene exfoliation in the polymer matrix are important factors influencing the gas transport [277]. In addition, the high mechanical strength, thermal stability and electrical conductivity allow excellent applications. However, the aggregation of graphene derivatives at high loadings, the local defects of nanocomposites during preparation and the good dispersability in the matrix are significant obstacles to overcome when preparing graphene-polymer nanocomposites.

As a further very positive perspective in terms of the use of graphene in packaging applications, graphene deposits were shown to have sufficient resilience to withstand thermoforming [286]. It was also demonstrated to extend the shelf life of beer packed in PET bottles by a factor of 2 to 5 [287]. In this study, diamond-like carbon (DLC) was deposited using a microwave plasma reactor to reach nanocoatings in the range of 50 nm thickness leading to over 10-fold decrease in the oxygen permeation. The optical properties of the coating were reported to vary from semi-transparent to fully transparent depending on the technology used.

##### Other Nanoparticles with Potential in Packaging Applications

Talc qualifies as good reinforcement filler of polymeric matrices because it is a layered mineral with a high aspect ratio (particle diameter/thickness ≈ 20:1). This is a consequence of its platy nature, having micron-sized dimensions on length and width, with nanometric thicknesses.

The size of an individual talc platelet (a few thousand of elementary layers) can vary from approximately 1 to over 100 µm, depending on the conditions of core formation. Van der Waals’ gaps (interlayer or gallery) between the layers are formed due to stacking, which may assist in the delamination behaviour of talc particles during the blending with a matrix. Layer charge is zero or very small, as there are not ions present between layers.

The effects of talc on synthetic polymers have been large studied. It was demonstrated that talc improves mechanical properties and macromolecular orientation of polypropylene [288]. Moreover, an induced crystalline structure has been reported, suggesting that talc particles act as a nucleating agent for polymer crystallization. Also, high aspect ratio platelets have been used to improve gas barrier properties.

Furthermore, even though this is not a primary goal, other nanoparticles such as pyrogenic silica have also been reported to improve the barrier properties of the matrices where they are dispersed [123]. Although non-platelet like nanoparticles lead to lower increase of tortuosity effect, their high specific surface area may lead to gas adsorption and above all, as here when dispersed in PP, they may act as nucleating agent increasing the crystallization degree of the matrix which is well known to increase barrier properties.

The electro-hydrodynamic processing (EHDP) was described in Section 2.2.2. Electrospraying is an efficient technique to develop nanostructured surfaces and incorporate nanofillers into the package polymer matrix, as shown in several published studies [83,289,290]. The use of EHDP is also very interesting for the incorporation of active substances (e.g., antimicrobials and antioxidants) within the package polymer matrix, for the development of active packaging [291,292].

#### 4.1.2. Easy-to-Empty Features

The adherence of liquids and other viscous products result in residues in packages, which leads to needless waste at the consumer end and difficulties in the packaging recycling. For reducing the residues in packages, the main objective consists of minimizing the interaction forces between the filled good and the food contact material of the packaging [293]. In Section 3.2.1, different methods to provide repellent properties have been described, by creating both hydrophobic or lipophobic surfaces. It has been shown that the wettability of a solid surface is governed by its surface energy determined by chemistry and texture [294]. A typical commercial example for anti-adhesive materials is Polytetrafluoroethylene (PTFE well-known under the tradename Teflon), usually used in non-stick cookware. The incorporating of fluorine atoms, which have a small atomic radius and high electronegativity, provide a low surface energy [295]. A smooth surface coated with densely packed fluorine atoms shows a water contact angle of approximately 120° [295], while by roughening such a surface, its water contact angle became greater than 150° [296]. However, the use of Teflon in packaging is hindered due to its high cost, high processing temperatures and low acceptability because of its fluorinated content.

Alternatively, low temperature surface modification processes have been described, since generally the polymers currently used in the packaging industry (PP, PE, PET …) are not heat-resistant. For example, ultra-water-repellent poly-(ethylene terephthalate) (PET) substrates have been fabricated by a two-step dry process. First, PET substrates were treated with oxygen plasma in order to provide a proper nanotexture, and subsequently a hydrophobic layer was coated on the nanotextured PET surfaces by means of either low-temperature chemical vapour deposition (CVD) using fluoroalkylsilane or plasma enhanced CVD using tetramethylsilane [297].

Recently, the Massachusetts Institute of Technology developed the first permanently wet slippery surface that can be used for easy-to-empty packaging (LiquiGlide). This solution is durable and makes viscous liquids slide easily [298]. The technology relies on non-wetting surface containing micro/nanotextures impregnated with lubricating liquids, which have been shown to exhibit superior non-wetting performance compared to superhydrophobic surfaces based on stable air-liquid interfaces [28,299]. For determination and comparison of the emptying behaviour, depletion or tack test methods can be applied for evaluation [300].

Although the development of non-wetting surfaces has been studied for a long time, easy emptying packaging is not a widely used option nowadays, due to the higher costs of the packages compared to traditional materials. However, the last improvements made in the nanomaterials field and the focus on high added value goods will pull the market for easy-to empty packaging solutions.

### 4.2. Solar Panels

Organic photovoltaic films have several advantages over conventional silicon cells. Photoactive organic materials are printed in extremely thin layers on transparent plastic film. The patented special inks used in printing consist of formulated blends of materials which after coating create electricity when exposed to light. This technology allows lightweight and flexible semi-transparent modules. As a result, they can be used on all kinds of surfaces. A further key advantage is that the modules generate relatively constant output, for instance even if it is cloudy or artificial light is being used. In addition, they can be produced in different colours and thus adapted to the surroundings. This is a property that creates new possibilities, particularly for building design. The cost-effective production of the OPV modules is also advantageous. Since these polymer materials can be processed as liquid solutions, they are suitable for multiple printing processes: spin coating, ink-jet printing or roll-to-roll processing such as gravure and flexographic printing [301].

There are many important advantages of OPV technology, especially with respect to building-integrated photovoltaics (BIPV). OPV modules do not show the performance drop usually observed with traditional inorganic photovoltaics in diffuse lighting conditions and under elevated temperatures—typical conditions found in façades. In addition, semi-transparency and tunable colours as well as freedom of design in shape and form are attractive and often even essential features for BIPV applications. Buildings account for 40% of energy consumption and 36% of carbon dioxide emissions in the EU. As a consequence, the EU has set a target for all new buildings to be nearly zero-energy (NZEB) as of 2021. The achievement of the legally binding NZEB objectives will require active building envelopes since passive materials are reaching their own limits. Gray OPV-based active building elements are an important step forward to combine energy generation and the aesthetic needs of architects [302].

Nanostructures on the front of the PV can guide light into the absorbing layer, or reduce reflection. Nanostructures on the back of a PV could be used as high performance reflectors, bouncing otherwise lost light back into the PV. The light-absorbing layer itself can benefit from a sculpted nanostructure, which could change its ability to absorb light of different wavelengths, for instance. Besides lower material costs, thin-film photovoltaics (tfPV) also are flexible because they only use very thin silicon, whereas current non-thin-film PVs are rigid. This could make tfPVs easier to install; like paper, they could be spooled off a roll [303,304].

Nanoparticles are established materials for OPV, but only mainly inside the OPV itself. Fullerenes are used as electron acceptor and electron transporting material [29]. PEDOT:PSS is used as a hole selective and transporting material [305,306,307,308]. As inorganic n-type contacts, TiO*_x_* and ZnO can be used. Also indium-doped zinc oxide (IZO) is possible [29]. An additional incorporation of metal nanoparticles such as gold or silver showed efficiency enhancement of polymeric solar cells, mainly ascribed to improved photocurrent density resulting from an excited localized surface plasmon resonance [309,310,311]. For the outer layer, properties like barrier and/or self-cleaning nanoparticles are developed not specifically for OPV but for solar cells in general. During their usage, OPV cells are exposed to several atmospheric degradation agents and thus they need to be protected by coatings and encapsulants. Nowadays, the following main properties are basically required for solar cells coating materials to ensure devices durability: UV, oxygen and water barrier; thermal stability, transparency, anti-reflectance, anti-soiling, flexibility, affordable cost, electrical isolation. The fouling due to dust, rains, bird faeces, etc., is a well-known issue in the industry that lead to loss of efficiency of solar panels with time. Surface-bound fog similarly scatters light and reduces optical transmission for transparent materials which is detrimental to their function [312]. The application of nanocoatings is also an interesting prospect for solar panels leading, in an easier way than the creation of organized nanostructured surfaces, to tailored repellence of liquid and other unwanted substances that deposit on the panel with time. Indeed, typical fouling (dust, dirt, rain, etc.) lead to significant losses of energy harvesting efficiency (up to 40%) and requires frequent maintenance. Therefore, a number of initiatives related to their self-cleaning are already on the market [313,314]. They employ coatings based on nanoparticles or texturation to get a lotus effect [315], or alternatively apply small electrical field to prevent dust from adhering on the surface. The Nanoshell^®^ technology claims it allows self-cleaning for up to five years and solar efficiency gains of up to 27% percent in wet weather since stationary rainwater on the panel limits the solar energy that is captured. Nonetheless, this later coating is applied manually post production, whereas electrospray seems an optimum route for the application of tailored coatings both on the point of view of reducing the amount needed to get a required effect (therefore saving cost) and of its easy integration within the existing process. Zhao and others described the electrospray deposition as a thin film deposition method that is uniquely suited for manufacturing organic photovoltaic cells with the desired characteristics of atmospheric pressure fabrication, roll-to-roll compatibility, less material loss, and possible self-organized nanostructures [316]. The main functionalities developed in the last reported studies focus on increasing the conductivity [317,318], and providing self-cleaning and anti-reflective properties [218] which contribute to extending the photovoltaic cells lifespan. Although a significant number of patents regarding self-cleaning solar panels in general (none for flexible OPVs though) were returned in representation of some of the above listed technologies, none of them employed electrospray as a deposition technique. Furthermore, based on nanostructured surfaces, researchers recently developed solar cells that can harvest light from any angle, and lead to self-cleaning panels at the same time [30].

Finally, inorganic nanocoatings and nanocomposites are well known to be transparent in the visible range of the spectra to allow harvesting useful light while filtering UV-light [319] potentially resulting in increasing the lifetime of the OPVs by preventing UV weathering.

All in all, the prospects of nano in solar energy are countless, and it has been one of the key drivers contributing to huge energy efficiency enhancement of the solar panels since their creation over 50 years ago.

### 4.3. Automotive Parts

The automotive sector can benefit from the utilization of nanomaterials. In this sense, the polymer nanocomposites can improve the performance of existing technologies in applications such as engines and powertrains, exhaust systems and catalytic converters, paints and coatings, tires, lighter but stronger materials, suspension and breaking systems, electric and electronic equipment, or frames and body parts [320].

On the one hand, the traditional fillers used in automotive parts (talc, mica and calcium carbonate) provide a higher stiffness, increasing melt viscosity and weight, and decrease the toughness and optical clarity. The glass fibre reinforcements introduce higher stiffness, but increased costs and difficulty of fabrication. Both glass fibre reinforcement and traditional fillers must be used at high loading to enhance the properties such as high modulus, or improve the dimensional stability, and the weight, toughness and surface quality are affected. 

On the other hand, the nanofillers such as nanoclays, are effective at lower concentrations (<5%). An improvement in modulus, fire retardancy, dimensional and thermal stability has been reported [321].

Nanoclays are the dominant commercial nanomaterials. It is as strong as 7% glass filled polyamide, with a density of 1.14 g/cm is an ideal light weight substitute for PA + 30% glass beads. Nanoclay replaces the traditional fillers at a 3:1 ratio.

Because of the low cost and enhanced physico-mechanical properties, polyolefins are becoming the most used thermoplastic in nanocomposites, exceeding earlier nylon 6/clay nanocomposites.

There is currently a growing interest to reduce the weight of the components in a car to reduce the fuel consumption. After the first nylon 6-clay nanocomposites were commercially used by Toyota in 1989 [6], General Motor followed in 2002, with a thermoplastic filled with 3% nanoclays for a step-assist. In 2004, the Chevrolet had a body side trim with nanoclays, and with a new design part, a weight saving of 25% was achieved. Another example with the same nanofiller was shown again for General Motors in 2005 to diminish the weight. Maserati engine bay covers were made with a nanocomposite of nylon 6 and nanoclays, reducing the weight, and increasing mechanical properties. 

Nowadays, Mucell (Microcellular foaming technology for injection moulding industry) is another process to reduce the weight in not structural components parts. It injects supercritical fluid in molten polymer to form microstructured foam with a solid skin layer and closed cells. As opposed to the use of nanofillers for reinforcement, this process leads to significant loss of mechanical properties and therefore of structural function. Therefore, nanofillers are still in development to reduce the weight with an improvement of other properties and a low cost.

The cost-performance ratio is the main objective for nanocomposites, due to the manufacturing cost of the nanoparticles. Besides, it is important to consider the change of design in the weight reduction with nanocomposites, as well as the improvement of material properties. The growth in research activity in terms of nanocomposites for the automotive sector continues to expand their applications. 

## 5. Nanosafety

As is evident in this review, there are many applications of nanotechnology that involve the use of nanoparticles in a variety of applications and products. This proliferation of nanotechnology has prompted concerns over potential risks from engineered nanoparticles where exposure to humans and/or the environment occurs intentionally or accidentally. The seminal report highlighting the opportunities and uncertainties of nanotechnologies and the potential risks posed by nanomaterials was published in 2004 by the UK’s Royal Society and the Royal Academy of Engineering [104]. This was one of the first reports to highlight the potential risks to health and the environment that may arise from exposure to nanomaterials. Since then, innumerable national and international reviews have provided a consistent view about the nature and the potential risks of nanoparticles, which may be summarised as follows:

- There are potential hazards to human health and the environment from certain types and forms of nanoparticles, but not all, and this is largely influenced by their composition and morphology;
- There is a paucity of knowledge about whether and how these potential hazards manifest as actual risks to human and environmental health, through exposure, and their significance;
- The absence of data makes it challenging for manufacturers, suppliers and users to have well-informed and effective risk management processes in compliance with their regulatory obligations.

Over the past decade, there has been a significant increase in research activity internationally, intended to fill these gaps. In Europe, this is particularly evident through the European Commission’s Framework Programmes. Outputs from this research are intended to further contribute to the field’s evidence base in a variety of ways. In risk assessment this through the assimilation of the wealth of scientific data on health and environmental implications of manufactured nanomaterials to be applied alongside knowledge of materials’ production, application and resulting potential new exposure pathways as part of responsible innovation. Several frameworks are available for assessing and managing risks from particulate nanomaterials, all of which are based on a common risk assessment approach. For example, the International Organization for Standardization (ISO) has proposed a step-by-step approach for nanomaterial risk evaluation and management [322].

Risk assessment is an integrative approach that considers the effects that potential hazards can have should exposure occur. Undertaking a risk assessment relies on having:

- good information about the hazardous nature of materials;
- good information about the effectiveness of control approaches;
- convenient and accessible ways to monitor exposure.

Exposure is characterized by measuring concentration and duration of exposure and this exposure analysis is important for risk assessment and subsequent risk management involving exposure control plans. In an occupational setting, exposure to nanomaterials can occur for workers at all phases of the material life cycle. During the development of a new material or process, it is likely that the material will be produced under controlled conditions, typically in small quantities, and although accidental releases, for example due to spills, are a possibility although in general, relatively few people are likely to be exposed at this stage. Once the material moves through pilot-scale into commercial production, more widespread exposures can potentially occur following the manufacture of the material or in downstream activities such as recovery, packaging, transport, and storage. Some materials may be subsequently incorporated into a range of other products or may be used in other processes as a feed-stock material. In these circumstances, the quantities of materials being handled can be expected to be much larger. 

Exposure to nanoparticles depends upon the formulation of nanoparticles during production, their use in products, and their potential release at during service life and at the point of recycling or disposal. Nanoparticles may be attached to surfaces (e.g., surface coatings), dispersed in solids (e.g., nanocomposites used for food packaging), suspended in liquids (e.g., TiO_2_ in sunscreen), as a powder, or be airborne (e.g., during production of nanoparticles). Suspensions of nanomaterials may represent a risk in terms of dermal exposure (see Poland et al. for a review [323]) and the solvent used for the preparation may also play an important role in influencing exposure if it can increase penetration of the material through the skin barrier or alter its size distribution for example. Suspensions of the materials are generally considered safer in terms of inhalation. However, care should be taken if physical processes such as centrifugation, ultra-sonication, heating and milling are applied to these suspensions which may release aerosols or dry material after solvent evaporation. 

In case of nanocomposites, the nanomaterial is fully embedded within a polymer matrix. Exposure to the nanomaterial would only be possible by migration of the nanomaterial out of the polymer (based on the Fickian law of diffusion) or by degradation of the polymer. In the first case it is believed that, once the nanomaterial is incorporated into a polymer matrix, it is immobilized wherefore migration cannot take place. This was examined for a variety of nanocomposites intended to be used as food packaging plastics. However, detection of possibly migrated nanomaterials in complex matrices, like food or food simulants, is quite challenging, wherefore the results of migration studies are often not consistent and sometimes even contradictory. A comprehensive overview on this topic can be found elsewhere [324,325,326].

The majority of studies dealing with the exposure during polymer-nanocomposites production have intended to replicate and assess particle release during the consumer/professional use stage of the nanocomposite lifecycle, often in a worst-case scenario, and may not involve the same operational conditions and risk management measures as would be present in an industrial situation. Nevertheless, they provide a first indication of the potential for nanoparticle release during the use of such methods, which could potentially be used during the finishing of nanocomposite articles. In order to obtain some preliminary information on potential release, researchers from the US National Institute for Occupational Safety and Health (NIOSH) undertook monitoring during the cutting of a nanocomposite paper containing graphene platelets, carbon nanotubes and other ingredients, using an unventilated band saw [327]. Real-time monitoring showed a very large increase in both particle number and mass concentrations when the composite was cut, with particles detected in the size range 7–100 nm. Although further research is needed to determine whether individual nanoparticles are being released, the authors recommend that if composites are routinely being cut by a band saw, dust control measures should be implemented. In a later study at the same workplace, Heitbrink, Lo et al. investigated the release of nanofibres during cutting and sanding of composite panels containing graphite fibres and/or carbon nanotubes [328]. A ventilated enclosure was built to capture and mix the emissions. Cutting the nanocomposite with a band saw did not result in the detection of carbon nanotubes or other fibres by transmission electron microscopy (TEM); however, the number concentration of particles <560 nm increased to over 105 particles/cm^3^ compared to background levels of 104 particles/cm^3^. The authors suggest that that high number concentration and emission rates may have been caused by the formation of nano-aerosols generated by frictional heating and did not appear to be elevated by the presence of carbon nanotubes. Sanding of a composite containing carbon nanotubes, generated fibre emission rates of 1.9 × 10^8^ and 2.8 × 10^6^ fibres/second, whilst measurable fibre concentrations were generated from panels that contained graphite and carbon fibres. The authors recommend the use of either a local exhaust ventilation hood or a high velocity, low volume ventilation system during such tasks. Numerous other studies have indicated the potential for significant particle release during the machining of carbon-based nanocomposites [329,330], most noticeably during dry surface grinding [331,332], dry cutting [333,334] or dry drilling [335] with the level of release being highly dependent on the sample material, sample composition, how well the nanomaterial is bound in the polymer matrix and the energy applied to the process. Performing the same tasks under wet conditions has been shown to be an effective method for reducing the number of airborne particles [333,335]. Based on the results of these studies, the release of matrix particles with and without embedded nanomaterials is common; however, the release of dissociated or “free” nanomaterials is rare [330,336,337,338]. This implies that, whilst degradation occurs during such tasks, nanomaterials often remain bound to the matrix. Manual sanding of carbon-based nanocomposites appears to result in lower particle emissions overall [336,339], with results indicating that particle release may be no higher than for sanding conventional composite materials. However, studies have indicated that micro-sized particles may be produced by abrasion, including with CNTs protruding from the main core [339]. Most recently, Schlagenhauf and colleagues have published two studies investigating CNT containing epoxy-based nanocomposites [337,338]. Their first study took a novel approach to the detection of CNT by labelling them with lead ions based on surface absorption before incorporation into an epoxy resin which was then abraded using a Taber Abraser. They found that in contrast to previous studies, the poorer dispersed CNT were released to a lesser extent than found with better dispersions. This was attributed to the relatively low energy of the abrasion process used which they surmised was not sufficient to break up CNT agglomerates. They were also able to predict that if 1 g of the nanocomposite had been abraded, 40 µg would be present as protruding CNT whilst there would be 0.4 µg of free CNT [337]. Given the potential for release, it has therefore been recommended that effective personal protection and engineering controls are used for all tasks involving the machining of nanocomposites, such as a ventilated enclosure equipped with HEPA filtration to prevent fugitive releases from contaminating the work area.

Further processing or simply aging of composites containing nanomaterials may lead to the release of particles and potential exposure. Organic polymers are particularly sensitive to UV radiation that can degrade them could lead to the release of particles. There are several studies which have evaluated the effect of sunlight (as well as other forms of weathering) on nanocomposites and one such study is that of Bernard et al. (2010) who investigated the fate of graphene oxide sheets in polymers under UV irradiation [340]. The results indicated that under UV irradiation, the polyurethane matrix showed signs of degradation. In parallel, accumulation of GFN at the surface of the composite was also recorded. A later study showed similar results after the UV weathering of an elastic CNT-polyurethane nanocomposite which contained 3% CNT and was further processed by injection moulding or extrusion before weathering through UV exposure [341]. They showed that dry weathering in a progressive way led to revealing of CNT as the matrix degraded but the nanofillers did not, leading to an accumulation at the surface. Indeed, further analysis showed that 72% of the top 10 nm of the weathered sample consisted of CNT. These results indicate that the choice of the matrix and its ageing under sunlight may lead to accumulation of nanomaterials at the surface which could influence releases to the environment [340].

An additional key issue within risk is the nature of hazards posed by nanocomposites and in particular, the nano-fillers used. There are relatively limited numbers of studies considering the toxicity of particles released from nano-composites and those which do exist tend to focus on carbon-based nanomaterials such as carbon nanotubes (CNT). Whilst these materials do present a rather polarised view of the use of nanomaterials in composites, they can be used to inform us as to how the toxicity profile of a nanomaterial may be altered by incorporation and subsequent release.

One such study is that of Wohlleben et al. (2011) which evaluated the respiratory toxicity of particulates released after abrasion of cement and thermoplastic based nanocomposites containing CNT. Animals were instilled with respirable (<10 µm) fractions of abraded cement or thermoplastic composites with or without carbon nanotubes and their toxicity compared to that of carbon nanotubes alone. The results showed that instillation of the matrix alone induced no clinical signs of toxicity or genotoxicity and minimal inflammation in the lung which was greater for the cement (a known respiratory irritant) yet instillation of the inhalable fraction of the nanocomposites did not lead to any differences in terms of toxicity compared to the matrix alone [336]. This was in stark contrast to instillation of carbon nanotubes at a four-fold lower dose which led to prominent lung inflammation which was abrogated by its inclusion into a composite. Similar results have been noted in a range of in vitro studies addressing CNT-polyurethane nanocomposites [341] as well as CNT containing epoxy-based nanocomposites [337,338].

Whilst these studies deal with a specific form of nanomaterial which is of course not representative of all nanomaterials (not least due to their fibrous nature which may influence release characteristics), they do show that incorporation into a composite can have a significant effect on toxicity. CNT are known to present a respiratory hazard and can induce inflammation as well as granuloma formation in the lung [342,343] and one form (not addressed within the studies above) has been classified as a possible carcinogen [344]. However, despite this intrinsic activity, the studies above show that incorporation into a variety of different composites is associated with a decrease in respiratory toxicity to the levels of the composite material alone meaning that the hazards associated with such nanocomposites may in fact be more akin to that of the base composite than the pure nanomaterial. Such observations are, however, based on a limited number of studies and do not consider many types of nanomaterials but if the overall principle noted is transferable (i.e., a net lowering of potential hazards) then this may be applied to other nanomaterials, particularly if they are of lower toxicity (e.g., TiO_2_).

The release of nanomaterials from coatings has been assessed in only a few studies, mainly testing the release of nanomaterials from paints and other coatings. Koponen et al. compared the effect of sanding on paints, with and without nanoparticles (carbon black and TiO_2_) [345]. The authors showed that although the geometric mean diameter of aerosol released during the sanding of paints was only slightly different with compared to without nanoparticles, the particle number concentration was increased during sanding of nanoparticle-containing paints [345].

To conclude, the safe use of chemicals and responsible development of processes and products are recognized as fundamental to ensuring a safe working environment through easy to implement, affordable and fit-for-purpose measures to mitigate any hazards and control exposure to nanomaterials, protecting the health of workers, consumers and the environment and supporting the commercialisation of nanotechnology for societal benefit. 

## 6. Conclusions

As seen during the last 20–30 years, significant progresses have been made in synthesis, processing, performances of polymer nanocomposites and nanocoatings. This article reviewed some key aspects on these fast-growing research areas in order to understand potential applications of polymer nanocomposites. These materials offer improved performance over bulk materials and microcomposites and hence can be used to overcome the limitations of many currently existing materials and devices. Nevertheless, a full control over their morphology (nanostructure dispersion or even orientation) is still desired to consistently reach optimal properties.

While polymer nanocomposite materials have unique behaviour such as improved mechanical and gas barrier properties, even upon small addition of nanofillers, nano-coatings can tailor the surface properties where they are applied for example in terms of affinity to liquids, but also of UV, gas or flame protection. Besides packaging, automotive and solar energy, nanocomposites’ applicative potential is endless; it includes bio-/chemical sensing, electronic devices, drug delivery, microwave absorbing device, orthopedic application, etc. 

There are different processes for the preparation of polymer nanocomposite materials each of each has its own advantages and drawbacks; therefore, the suitable methods should be adjusted to the target application, composition, dispersion performance, etc. In terms of process improvements, ultrasonic-assisted dispersions have shown some encouraging results both in liquid media and melt plastic stream. This later also faces questioning related with the nano-safety of workers when handling nanoparticles, as well as with their use within consumer applications. While for specific nanoparticles, the amount of research performed allows us to confirm the absence of hazards, the number of parameters involved is so high that generalization to others should be avoided.

## Figures and Tables

**Table 1:** Models for predicting barrier properties of platelet filled nanocomposites [8].

Model	Filler Type	Particle Geometry	Formulas	Reference
Nielsen	Ribbon ^a^	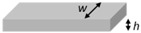	(*P*_0_/*P*)(1 − φ) *=* 1 *+* αφ/2	[134]
Cussler (Regular array)	Ribbon ^a^	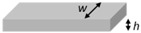	(*P*_0_/*P*)(1 − φ) *=* (1 *+* αφ)^2^/4	[135]
Cussler (Random array)	Ribbon ^a^	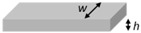	(*P*_0_/*P*)(1 − φ) *=* (1 *+* αφ/3)^2^	[135]
Gusev and Lusti	Disk ^b^	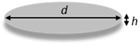	(*P*_0_/*P*)(1 − φ) *= exp*[(αφ/3.47)^0.71^]	[136]
Fredrickson and Bicerano	Disk ^b^	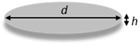	(*P*_0_/*P*)(1 *−* φ) *=* 4(1 *+ x +* 0.1245*x*^2^)/(2 *+ x*)^2^ *where x =* αφ/2*ln*(*α*/2)	[137]
Bharadwaj	Disk ^b^	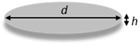	(*P*_0_/*P*)(1 *−* φ) *=* 1 *+* 0.667αφ(*S +* (1/2)) *where S = orientation factor* (*from −*1/2 *to* 1)	[138]

^a^ For ribbons, length is infinite, width, *w*; thickness, *t*; aspect ratio, *a* = *w*/*h*; ^b^ For disks, circular shape of diameter *d* and thickness *t*; aspect ratio, *a* = *d*/*h*.

**Table 2:** Glass transition changes with nano-filler incorporation. SWCNT = single-walled carbon nanotubes; MMT = montmorillonite; MWCNT = multi-walled carbon nanotubes.

Polymer	Nanofiller	*T*_g_ Change	References
**Polystyrene**	SWCNT	3	[157]
**Polycarbonate**	SiC (0.5–1.5 wt %) (20–60 nm particles)	No change	[159]
**Poly(vinyl chloride)**	Exfoliated clay (MMT) (<10 wt %)	1 to 3	[160]
**Poly(dimethyl siloxane)**	Silica (2–3 nm)	10	[161]
**Poly(propylene carbonate)**	Nanoclay (4 wt %)	13	[162]
**Poly(methyl methacrylate)**	Nanoclay (2.5–15.1 wt %)	4–13	[163]
**Polyimide**	MWCNT (0.25–6.98 wt %)	4 to 8	[164]
**Polystyrene**	Nanoclay (5 wt %)	6.7	[165]
**Natural rubber**	Nanoclay (5 wt %)	3	[167]
**Poly(butylene terephthalate)**	Mica (3 wt %)	6	[168]
**Polylactide**	Nanoclay (3 wt %)	1 to 4	[158]

**Table 3:** Examples of polymer-clay nanocomposites and their barrier improvements. Permeabilities are expressed as improvement ratios: the ratio of the gas permeability or transmission rate of the virgin polymer to the gas permeability or transmission rate of the polymer-clay composite, measured at the same conditions [133].

Polymer Matrix	Filler	Clay (wt %)	*P*(O_2_)	*P*(H_2_O)	References
**PS**	Modified montmorillonite	16.7	2.8		[264]
**PET**	Modified montmorillonite	5	15.6	1.2	[265]
	Modified montmorillonite	5	2.23	1.15	[266]
**EVOH**	Kaolinite	5	3–4	1.2	[267]
**PLA**	Montmorillonite	5	1.16	1.21	[266]
	Modified montmorillonite	5	1.2–1.9	1.7–2	[268]
	Mica	4	2.8		[269]
**PHB**	Kaolinite	5	1.26	1.06	[266]
**HDPE**	Modified montmorillonite	4	1.2–1.7		[270]
	Modified montmorillonite	5	2.8–2.9	1.8–2.4	[271]
**LDPE**	Modified montmorillonite	4.76	2.2		[272]

**Table 4:** Representative examples of graphene-based nanocomposites targeting to improve gas barrier properties [276].

Polymer Matrix	Type of Graphene	Preparation Method	Maximum Fraction	Reference
**PS**	GO	Melting	2.27 vol %	[280]
**LLPDE**	Functionalized graphene	Solution mixing	5 wt %	[281]
**PET**	Functionalized GO	Solution mixing	3 wt%	[282]
	Reduced GO	Melting	1.5 wt%	[283]
**PLA**	GO, graphene	Solution mixing	0.6 wt%	[284]
**PP**	Reduced GO	Melting	1 wt %	[285]
